# Sequential formation of *Drosophila* circuit asymmetry via prolonged structural plasticity

**DOI:** 10.1126/sciadv.aea6020

**Published:** 2026-03-25

**Authors:** Johann W. Markovitsch, Daniel Mitić, Alisa del Pilar Jiménez García, Zane Alsberga, Sarah Kainz, Rashmit Kaur, Thomas Hummel

**Affiliations:** ^1^ Department of Neurosciences and Development, University of Vienna, Djerassiplatz 1, 1030 Vienna, Austria.; ^2^ Institute of Science and Technology Austria (ISTA), Am Campus 1, 3400 Klosterneuburg, Austria.; ^3^ Brain Research Institute, University of Zurich, Winterthurerstrasse 190, CH-8057 Zurich, Switzerland.

## Abstract

Structural and functional differences between brain hemispheres are a common feature of animal nervous systems with reduced bilateral asymmetry often linked to impaired cognitive performance. How neuronal left-right asymmetry is initiated and integrated into a bilaterally symmetrical ground pattern is poorly understood. Here, we show that the directional asymmetry of a *Drosophila* central brain circuit originates from axonal interactions of two types of bilateral pioneer neurons. Subsequent recruitment of neighboring neurons into the asymmetric neuropil primordium results in hemisphere-specific microcircuits. Circuit lateralization requires dynamic expression of the cell adhesion molecule Fasciclin 2 to maintain structural plasticity in axonal remodeling. Reduced circuit asymmetry following cell type–specific Fasciclin 2 manipulation affects adult brain function. These results reveal an unexpected degree of developmental plasticity of late-born *Drosophila* neurons in the formation of a circuit node via the lateralized recruitment of symmetric circuit components.

## Teaser

Late-born neurons in the developing *Drosophila* brain induce and integrate left-right asymmetry into bilateral neural circuits.

## INTRODUCTION

In bilaterian animals, the central nervous system develops with overall mirror symmetry that is maintained in adult neuronal organization. Although bilateral symmetry is widely assumed to have evolved in response to selection for directed locomotion (*1*), accumulating evidence indicates that bilateral brain organization, manifested in paired structures such as the vertebrate hemispheres, is accompanied by functional specialization between left and right homotopic regions. This phenomenon, termed lateralization, appears to have evolved independently multiple times, irrespective of brain size, suggesting a selective advantage of neuronal left-right asymmetry (*2*, *3*). Recent experimental data suggest that lateralization enhances cognitive performance (*4*–*6*), with reduced asymmetry negatively correlated with behavioral outcomes (*7*–*9*). Consistent with these findings, the link between information processing in the human brain and cerebral lateralization is supported by observations of reduced left-right asymmetry in schizophrenia, dyslexia, and autism spectrum disorders (*10*). The formation of lateralized neuronal circuits necessitates the incorporation of left-right asymmetric elements into an otherwise bilaterally symmetrical neural ground pattern. However, the developmental and circuit-level mechanisms underlying this process are not well understood.

The *Drosophila melanogaster* brain contains a bilateral synaptic neuropil, the asymmetrical bodies (ABs) (*7*, *11*), with left-right differences in volume (*11*), neuronal composition (*11*), and protein expression (*7*, *9*). A reduction of AB asymmetry correlates with deficits in olfactory (*7*, *9*) and courtship (*9*) long-term memory, indicating a direct relevance of central nervous system lateralization for cognitive function. The AB is embedded in a series of midline neuropils called the central complex (CX), which in addition encompasses the ellipsoid body (EB), the fan-shaped body (FB), the noduli (NO), and the protocerebral bridge (PB) (*11*, *12*). Although born as early as in the embryo (*13*), CX neuronal projections remain undifferentiated neural processes during the larval stages, making the CX functionally a structure of the adult organism (*14*). Lateralization and the formation of synaptic connection in the immature neuropil primordia occur much later during pupal development (*9*, *15*), making the CX a suitable experimental system for investigating symmetry breaking and asymmetric circuit reconfiguration.

Here, we show that the developmental interaction between two types of pioneer afferent neurons initiates lateralized remodeling of the AB neuropil, in which the dynamic expression of *Fasciclin 2* (*Fas2*) controls the level of type-specific asymmetry. Lateralized AB circuit assembly within the otherwise symmetric CX is mediated by the recruitment of early-born FB neurons to induce side-specific synapse formation. This degree of structural plasticity in building neuronal connections across brain neuropils is rather unique at this advanced state of *Drosophila* adult nervous system development and reveals brain lateralization as an additional genetically programmed mechanism for reorganizing the bilateral ground pattern.

## RESULTS

### Morphological and synaptic left-right asymmetry in CX connectivity

**Fig. 1.**
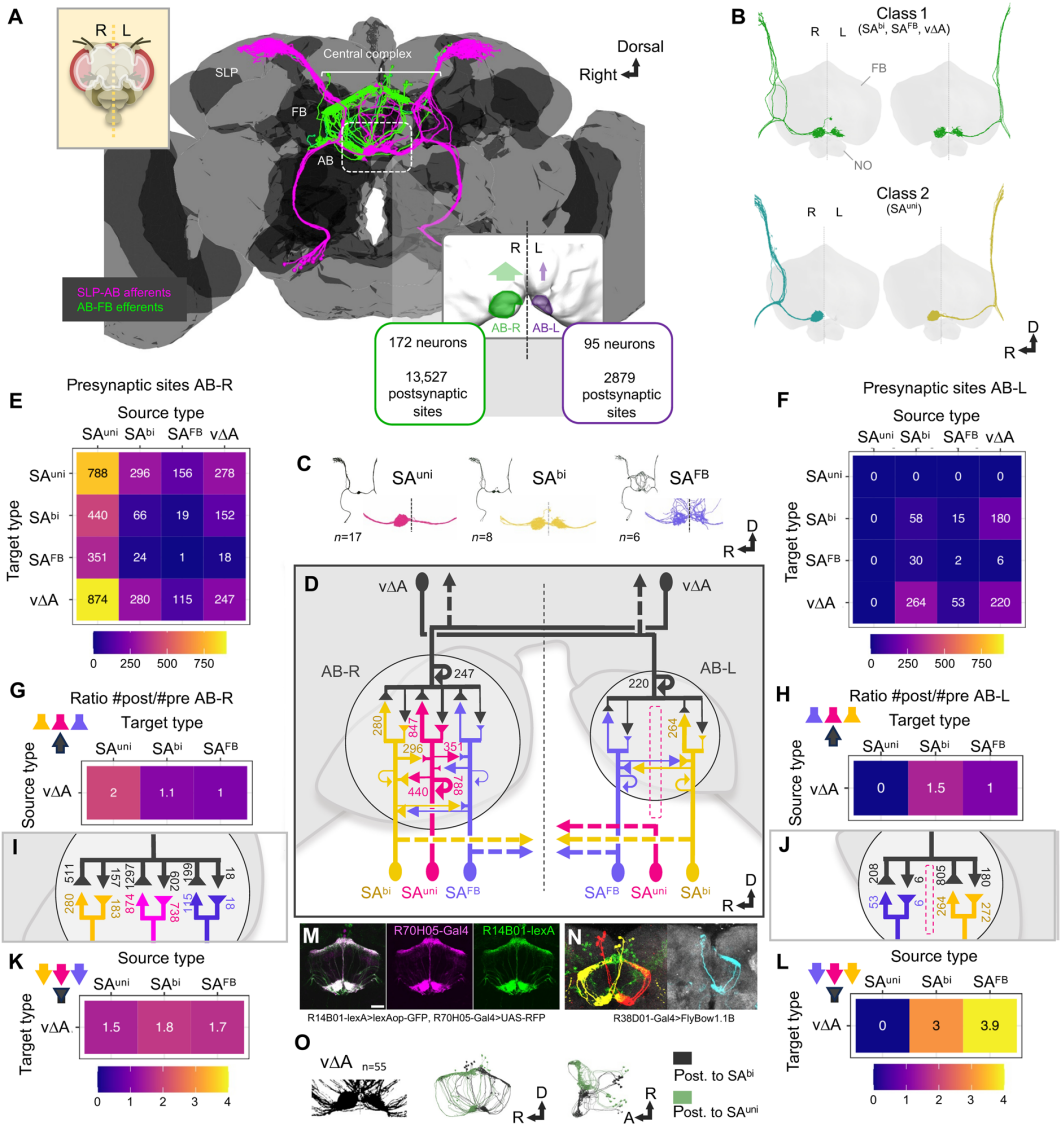
AB neuronal types showed different levels of interhemispheric asymmetry. (**A**) Position of the AB circuit in the adult *Drosophila* central brain [3D reconstruction from EM data, Flywire (*19*–*21*, *71*)] and left-right asymmetry in synaptic connections in the AB neuropils [EM data, Hemibrain (*16*, *18*)]. Afferent neurons in magenta and efferent neurons in green. (**B**) Two classes of morphological asymmetry of AB neurons: Neurons displayed a bilateral connectivity pattern but differ in the innervation between AB-R and AB-L (class 1); neurons show unilateral connectivity to the same hemisphere (AB-R) (class 2). 3D reconstruction from EM data (Hemibrain). FB and NO neuropils in gray. (**C**) 3D reconstruction from EM data (Hemibrain) for three main AB afferent neuron types, the unilateral axon projections of SLP-AB-unilateral (SA^uni^) compared to the two bilateral neuron types SLP-AB-bilateral (SA^bi^) and SLP-AB-FB (SA^FB^). Total cell numbers (*n*) for each SA type in both hemispheres (Hemibrain). (**D**) Schematic of the AB circuit and number of presynaptic sites targeting indicated neuron types (arrowheads) in AB-R and AB-L (Hemibrain). (**E** and **F**) Connectivity matrix for AB principal neuron types in AB-R (E) and AB-L (F). (**G** to **L**) Numbers and ratios of presynaptic sites and connected postsynaptic sites for SA^uni^ (magenta), SA^bi^ (yellow), SA^FB^ (blue), and v∆A (dark gray) in AB-R (G, I, and K) and AB-L (H, J, and L). (**M** and **N**) Expression lines and mosaic analysis of AB efferent neurons v∆A, which connect the AB with the dorsal FB. (M) Immunofluorescence detection of *lexAop-GFP* driven by *R14B01-lexA* and *UAS-RFP* driven by *R70H05-Gal4* (magenta). (N) MCFO-induced v∆A mosaic clones visualizing the projection of bilateral subclusters. (**O**) 3D reconstruction of all v∆A neurons (Hemibrain data) illustrating the differential input of SA^bi^ in AB-L (black) and SA^uni^ in AB-R (green).

Lateralization of the AB, a bilateral neuropil at the ventral border of the FB, can be visualized by morphology and synaptic connectivity of afferent and efferent neurons (Fig. 1 and fig. S1). The AB neuropil is highly interconnected with the FB and the “superior lateral protocerebrum” (SLP) in the *Drosophila* protocerebrum (*16*) via SLP-AB afferent neurons and AB-FB output neurons (Fig. 1, A and M). Three morphology types of AB afferent neurons, SLP-AB-unilateral (SA^uni^/SA1&2), SLP-AB-bilateral (SA^bi^/SA3), and SLP-AB-FB (SA^FB^/SAF), provide tangential synaptic input to the AB neuropil (Fig. 1, B and C; also see Materials and Methods for the nomenclature and connectome analysis). The cell bodies of all SA neuron types colocalize in the anterior-ventral cluster LALv1 (*17*) and extend in a common tract dorsally, which bifurcates into a dorsal SLP process and a horizontal CX process (Fig. 1, A and B). Lateralization of AB neuron connectivity is organized as either bilateral asymmetry with differences in the number of processes and synapses (class 1) or unilateral asymmetry without any innervation in one hemisphere (class 2) (Fig. 1, B and C). Compartment-specific marker expression (*Syt::GFP*, *Brp:GFP*, *DenMark::RFP*) revealed a polarized SA neuron organization with main dendritic domains in the SLP and a colocalization of pre- and postsynaptic sides within the AB domain (fig. S1, A and B). While their dendritic domains mostly overlap in the SLP region, the three types of afferent neurons differ in their presynaptic innervation between the AB neuropils in the right versus left hemisphere (Fig. 1, B and C, and fig. S1, C to H). The class 2 asymmetry of SA^uni^ defines the central connectivity node in the AB in the right hemisphere (AB-R) (Fig. 1, C and D). Because of the larger number of cells compared to other AB afferents (Fig. 1C), SA^uni^ neurons are the main synaptic input to v∆A (Fig. 1, D to F). In addition, SA^uni^ is a central component of afferent interconnectivity, not only toward SA^bi^ and SA^FB^ but also among SA^uni^ neurons (Fig. 1, D as overview and G and I as connectivity matrices) (*18*). Its capacity as an SLP-FB circuit hub is also reflected in the fact that about 60% of SA^uni^ postsynaptic sites are within AB-R (fig. S1G). This interconnectivity between AB afferents appears to be specific to SA^uni^ neurons, as SA^bi^ and SA^FB^ neurons form only sparse synapses with each other, both in AB-R and in the AB in the left hemisphere (AB-L), where SA^uni^ processes are absent (Fig. 1, D to F), indicating a distinct ability of SA^uni^ to organize synapse formation among AB afferent neurons.

Cell bodies of v∆A, the main AB relay neurons to the dorsal FB (Fig. 1A, green), form four spatially separated clusters per hemisphere (fig. S1, L and M), which reflect their descent from the DM1 to DM4 cell lineages (*19*–*21*). Their fibers follow the described projection pattern (*13*), with the v∆A neurons derived from DM1 and DM2 projecting contralaterally and v∆A neurons derived from DM3 and DM4 connecting AB and FB ipsilaterally (Fig. 1N and fig. S1, L and M). Most individual v∆A neurons innervate only AB-R or AB-L in both the Hemibrain dataset and our mosaic analysis, with a small fraction (of all four lineages) displaying bilateral AB-R/L connectivity via the commissural tract of SA afferents (Fig. 1N and fig. S1, R and S). Electron microscopy (EM) data revealed left-right asymmetry in synaptic differentiation of these bilaterally innervating v∆A neurons with an increase in postsynaptic sites in AB-R (fig. S1S), indicating a higher level of morphological plasticity compared to the stereotype pattern of CX columnar neurons (*16*).

With the absence of SA^uni^ axon terminals, AB-L contains fewer synapses compared to AB-R (Fig. 1, D to F, I, and J, and fig. S1, C to H, N, and P). The presynaptic sites of SA^bi^ onto v∆A appear evenly distributed between AB-L and AB-R (Fig. 1, D to H). While both SA^FB^ and v∆A neurons develop less postsynaptic sites in AB-L compared to AB-R (fig. S1, P and H), v∆A redistributes its pool of postsynaptic sites to different synaptic partners, particularly SA^bi^ (compare Fig. 1, I and K, with Fig. 1, J and L). As a result, the synaptic weight of single SA^bi^ presynaptic sites is higher in AB-L than AB-R (Fig. 1, K and L), resulting in switch in asymmetry direction (AB-R < AB-L) with roughly double the number of v∆A postsynaptic sites in the left hemisphere (Fig. 1J). Thus, the right dorsal FB in AB receives predominantly input from SA^uni^ neurons, while the left dorsal FB receives input from SA^bi^ (Fig. 1O). In summary, defined structural asymmetry in bilateral morphology and synaptic connectivity for all principal AB neuron types specifies right-directed lateralization in interhemispheric circuit organization.

### Cellular identity of AB afferent asymmetry

**Fig. 2.**
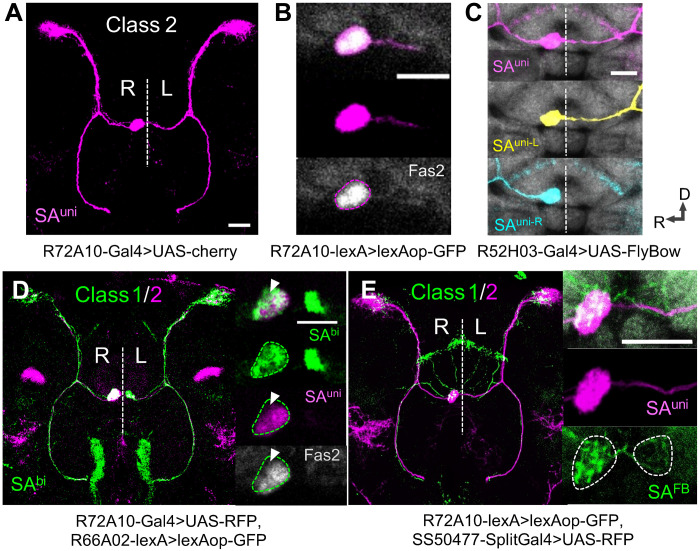
Two classes of interhemispheric asymmetry determine lateralized AB input. (**A**) Immunofluorescence detection of *UAS-cherry* driven by *R72A10-Gal4* (SA^uni^). (**B**) Innervations of AB-R by SA^uni^ overlap with the unilateral Fas2 expression (gray, antibody staining). (**C**) Afferent SA^uni^ projections are hemisphere-dependent (class 2 asymmetry): ipsilateral SA^uni-R^ (yellow) and contralateral SA^uni-L^ (blue). (**D**) SA^bi^ afferent neurons (green) project bilaterally to AB-R and AB-L with different innervation sizes (class 1 asymmetry). Fas2 expression (gray) fully overlaps with SA^uni^ in AB-R but is absent in SA^bi^ innervations of the dorsal AB-R (arrowhead; the dashed line indicates SA^bi^) and in AB-L. Immunofluorescence detection of *UAS-RFP* driven by *R72A10-Gal4* (SA^uni^, magenta), *lexAop-GFP* driven by *R66A02-lexA* (SA^bi^, green), and anti-Fas2 antibody staining (gray). (**E**) Bilateral afferent SA^FB^ neurons (green) project both to the dorsal FB and AB-R/AB-L with strong class 1 asymmetry (dashed lines). Immunofluorescence detection of *lexAop-GFP* driven by *R72A10-lexA* (SA^uni^, magenta), *UAS-RFP* driven by *SS50477-Split-Gal4* (SA^FB^, green), and CadN antibody staining. All scale bars, 20 μm.

The lack of AB class 2 asymmetry distinguishes SA^bi^ and SA^FB^ from SA^uni^, with SA^FB^ showing an additional innervation in the dorsal FB (Fig. 2). SA^uni^ and SA^bi^ appear morphologically almost identical, especially in the bilateral variant of SA^uni^, which can be detected in about 8% of wild-type *Drosophila* (*7*), raising the question whether SA^bi^, as described in the connectome, defines a distinct cell type (*20*). Because of class 2 asymmetry in neuronal morphology, SA^uni^ can be identified by the unilateral expression of the cell adhesion molecule Fas2 (Fig. 2B) (*7*). We found SA^bi^ neurons (labeled by *R66A02-Gal4/-lexA*) present in all brains bilaterally innervating AB-L and AB-R independent of the SA^uni^ projection pattern (Fig. 2D). While both uni- and bilateral morphotypes of SA^uni^ express Fas2, adult SA^bi^ neuronal processes in AB-R did not fully overlap with Fas2 antibody staining and were Fas2-negative in AB-L (Fig. 2D). These results confirm the Hemibrain annotation of SA^uni^ and SA^bi^ as two AB afferent neuron types with unique morphological and molecular features.

**Fig. 3.**
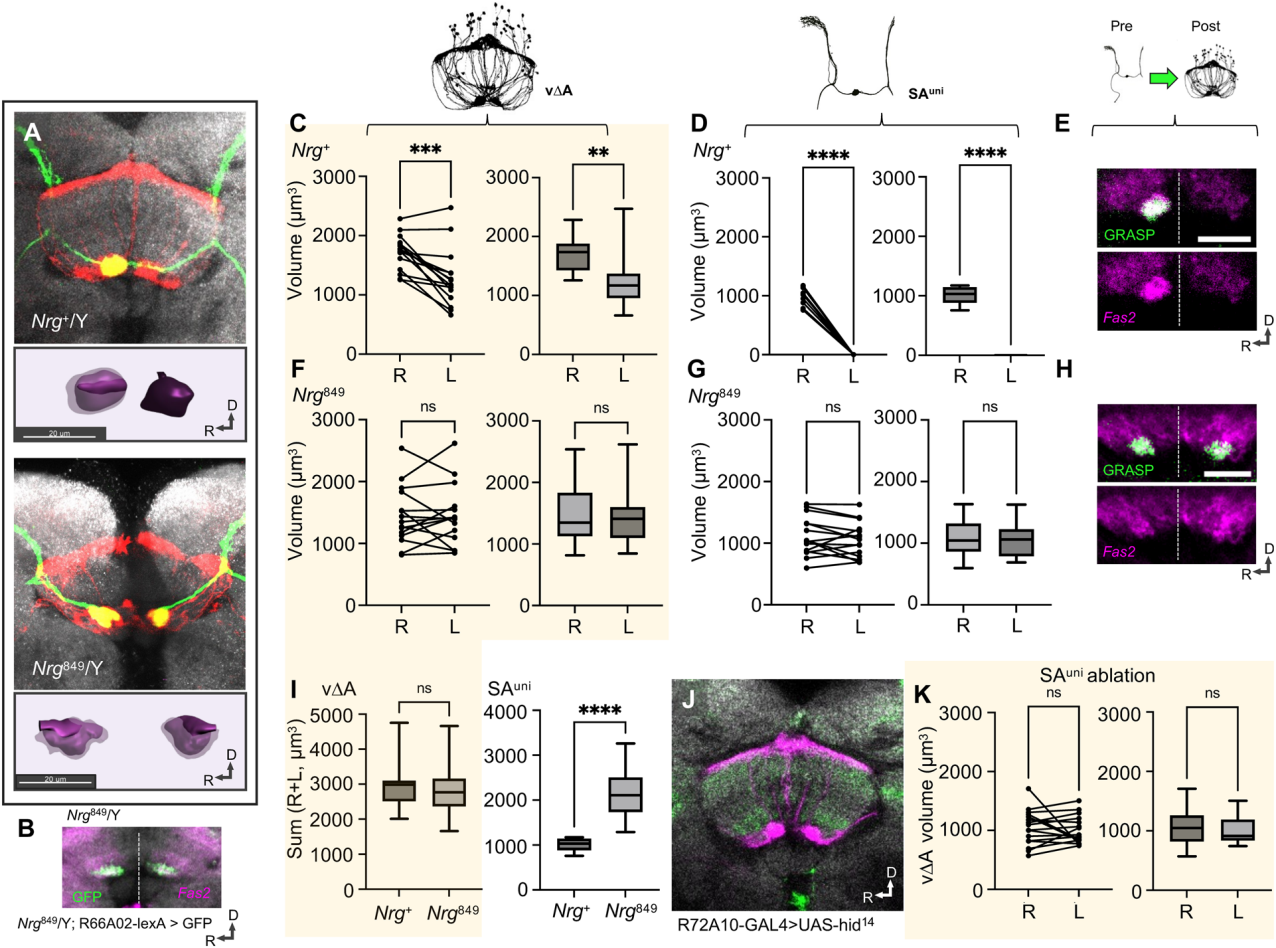
Lateralized AB innervation exhibits developmental plasticity. (**A**) AB innervation volumes were measured from immunodetection of *lexAop-GFP* driven by *R72A10-lexA* (SA^uni^) and *UAS-RFP* driven by *R70H05-Gal4* (v∆A). Hemizygosity for the hypomorphic allele *Nrg^849^* prevents SA^uni^ neurons [as well as SA^bi^ (**B**), *R66A02-lexA* driving *lexAop-GFP*] from forming commissures across the midline, while decussating projections by v∆A neurons are not affected. In wild-type brains, the volume of innervations by v∆A neurons (**C**) and SA^uni^ (**D**) is significantly larger in AB-R than in AB-L. SA^uni^ neurons in all measured brains innervated only AB-R. (**E**) Immunodetection of reconstituted GFP (green) from *nsyb-GRASP* driven by *R72A10-Gal4* (presynaptic) and *R38D01-lexA* (postsynaptic) and Fas2 (magenta) and CadN antibodies (gray) indicates functional synapses between SA^uni^ and v∆A in AB-R. Population-level volume differences of v∆A (**F**) and SA^uni^ (**G**) innervations between AB-R and AB-L were not detectable in *Nrg^849^* mutants. ns, not significant. (**H**) GRASP showed functional and active synapses between *Nrg*-induced ipsilateral SA^uni^ neurons and v∆A in AB-R and AB-L. Immunodetection of reconstituted GFP (green) from *nsyb-GRASP* driven by *R72A10-Gal4* (presynaptic) and *R38D01-lexA* (postsynaptic) and Fas2 (magenta) and CadN antibody (gray) staining. (**I**) The total AB volume innervated by SA^uni^ is twice as large in *Nrg*-induced ipsilateral neurons compared to the control. (**J**) The innervation pattern of v∆A in the CX following ablation of SA^uni^ through expression of *UAS-hid^14^* driven by *R72A10-Gal4*. Immunodetection of *lexA-opGFP* (magenta), driven by *R70H05-lexA* (v∆A), and CadN antibody staining (gray). (**K**) Following the ablation of SA^uni^ neurons, the left-right asymmetry of v∆A neuronal innervation was no longer detectable. Paired data plot (Wilcoxon test for paired samples) to compare the relationship of AB-R and AB-L measured from the same brain and box plot to compare distribution of measured innervation volumes between AB-R and AB-L (Mann-Whitney *U*). All scale bars, 20 μm. Significance thresholds are as follows: **P < 0.01; ***P < 0.001; ****P < 0.001.

While differences in the relative volume of axon arborizations between AB-R and AB-L are found for all AB afferents (Fig. 2), class 2 asymmetry of SA^uni^ is unique among neurons in the *D. melanogaster* brain, as each hemisphere contains a distinct morphological subtype, ipsilateral SA^uni-R^ and contralateral SA^uni-L^ neurons (Fig. 2C). To test whether SA^uni-R^ and SA^uni-L^ neurons are intrinsically different in organizing AB lateralization, we prevented contralateral projections across the midline in a hypomorphic *Neuroglian* mutant background (*Nrg^849^*) while leaving the overall SA^uni^ projections unaffected (Fig. 3, A and B). Both SA^uni-R^ and SA^uni-L^ neurons innervate the ipsilateral AB neuropil, and no directional size differences could be detected (compare Fig. 3, C to E, with Fig. 3, F to H). Induction of apoptosis in SA^uni^ had similar effects on v∆A volume [confirming results in (*15*)] (Fig. 3, J and K). Furthermore, ectopic ipsilateral SA^uni-L^ neurons form functional synapses with postsynaptic v∆A neurons (Fig. 3, E and H). No size differences in AB innervation of SA^bi^ can be detected in the *Nrg* mutant background (Fig. 3B). These results indicate a critical role of SA^uni^ in overall CX left-right asymmetry despite no obvious intrinsic differences between SA^uni-R^ and SA^uni-L^. Notably, in *Nrg* mutants, both SA^uni-L^ and SA^uni-R^ expand to volumes that match the combined wild-type innervation of AB-R, resulting in bilateral AB innervation that is doubled in size (Fig. 3I). As a disruption of interhemispheric connectivity prevents CX lateralization, we further explored the developmental profile and interactions of uni- and bilateral afferent neurons.

### Dynamic Fas2 expression during lateralized circuit remodeling

**Fig. 4.**
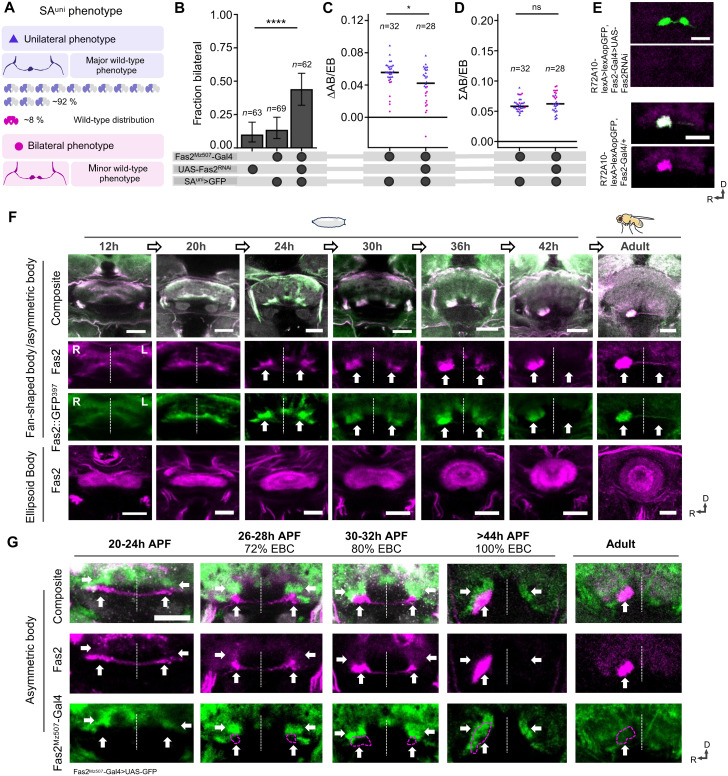
Dynamic Fas2 expression supports asymmetric AB development. (**A**) Schematic of the wild-type distribution of the two SA^uni^ morphotypes [major class 2 asymmetry (92%) and minor class 1 asymmetry (8%)]. (**B**) Targeted RNAi knockdown of Fas2 via *Fas2^Mz507^-Gal4* at 25°C increased the rate of brains with bilateral SA^uni^ connectivity (χ^2^). (**C**) Lateralization of innervation area was significantly decreased in the Fas2 knockdown group (∆AB/EB, Mann-Whitney *U*). (**D**) Brains with bilateral SA^uni^ connectivity tended to have a bigger overall AB innervation area (ΣAB/EB). (**E**) Fas2 antibody staining was no longer detectable in the AB. Immunofluorescence detection from *lexAop-GFP* (green) driven by *R72A10-lexA* and anti-Fas2 antibody staining (magenta). (**F** and **G**) Expression of Fas2 during AB development. Pupa brains were dissected at the indicated developmental time (hours APF). h, hours. ellipsoid body closure (EBC). (F) The gene trap line *Fas2::GFP^397^* (green) recapitulated endogenous Fas2 protein expression (magenta). (G) *Fas2^Mz507^-Gal4*–driven GFP expression labeled the dorsal domain of the AB primordium (horizontal arrows) but not the ventral AB domain (vertical arrows). *Fas2^Mz507^-Gal4*–driven GFP expression declined after AB remodeling (>44 hours APF) and was undetectable in the adult AB. Immunofluorescence of *UAS-GFP* driven by *Fas2^Mz507^-Gal4* (green), anti-Fas2 (magenta), and anti-CadN (gray) antibody staining. All scale bars, 20 μm. Error bars indicate two-sided 95% CIs for the single proportions. Blue triangles indicate measurements from brains with a unilateral AB projection by SA^uni^, and magenta dots indicate brains with a bilateral projection. Significance thresholds are as follows: *P < 0.05; ****P < 0.001.

The distinct expression of Fas2 in mature SA^uni^ neurons and the reported role of Fas2 in synaptic maturation and remodeling (*22*–*24*) suggest a function in the development of AB connectivity. Targeted RNA interference (RNAi) of Fas2 expression under the control of endogenous Fas2 enhancers (*Fas2^Mz507^-Gal4*, *Fas2^MiMIC12989^-Gal4*) resulted in the loss of anti-Fas2 antibody labeling [all transmembrane isoforms via 1D4 (*25*)] (fig. S2A) accompanied by a significant increase in bilateral SA^uni^ connectivity (Fig. 4, A to E), indicating a critical role of Fas2 in CX lateralization (see Materials and Methods for protocols of RNAi experiments, phenotype classification, and quantification of innervation area).

We next analyzed Fas2 expression during CX neuropil formation combining anti-Fas2 antibody labeling with an endogenously green fluorescent protein (GFP)–tagged Fas2 allele [*Fas2*^*GFP39*7^ (*25*)]. Throughout pupal development, we observed a close overlap between anti-Fas2 antibody labeling and Fas2^GFP^ fluorescence signals (Fig. 4F). At 12 hours after puparium formation (APF), a thin bundle of Fas2-positive commissural fibers at the ventral surface of the CX primordia could be detected, which increased in thickness until 20 hours APF when a bilateral pair of AB neuropils started to segregate from the ventral FB (Fig. 4F and fig. S2B). At 25 hours APF, small fluctuating asymmetry in the size of these AB primordia could be observed, but no consistent directional lateralization of Fas2 expression was evident (Fig. 4F and fig. S2B). Over the next 10 hours, a relative volume increase in aggregated Fas2-positive neuronal processes in the right hemisphere accompanied by an increase in Fas2-reporter expression could be observed (Fig. 4F and fig. S2B). In addition, within the AB-R primordium, Fas2 expression in the ventral half is increased compared to the dorsal domain (Fig. 4F). In most analyzed brains, the Fas2 signal in AB-L disappears around 40 hours APF, with rare extended expression until 45 hours APF (Fig. 4F and fig. S2B).

Next, we compared the expression of the two Fas2 Gal4 driver lines, which induced a bilateral SA^uni^ phenotype, *Fas2^Mz507^-Gal4* (Fig. 4G) and *Fas2^MiMIC12989^-Gal4* (fig. S2C) with Fas2 protein expression. Neither Fas2-Gal4 line drove reporter expression in the adult AB (Fig. 4G and fig. S2C). During pupal development, both Fas2 Gal4 lines labeled neurons that innervate only in the dorsal AB precursors, while both dorsal and ventral areas are Fas2-positive (Fig. 4G and fig. S2C). The decline of Gal4 expression coincided with Fas2 protein no longer detectable in AB-L. These results indicate that the early AB neuropil is formed by two molecularly distinct populations of afferent neurons, which segregate their axons along the dorsoventral axis of the neuropil primordium.

### Recruitment of symmetrical CX neurons into the asymmetric AB neuropil primordium

**Fig. 5.**
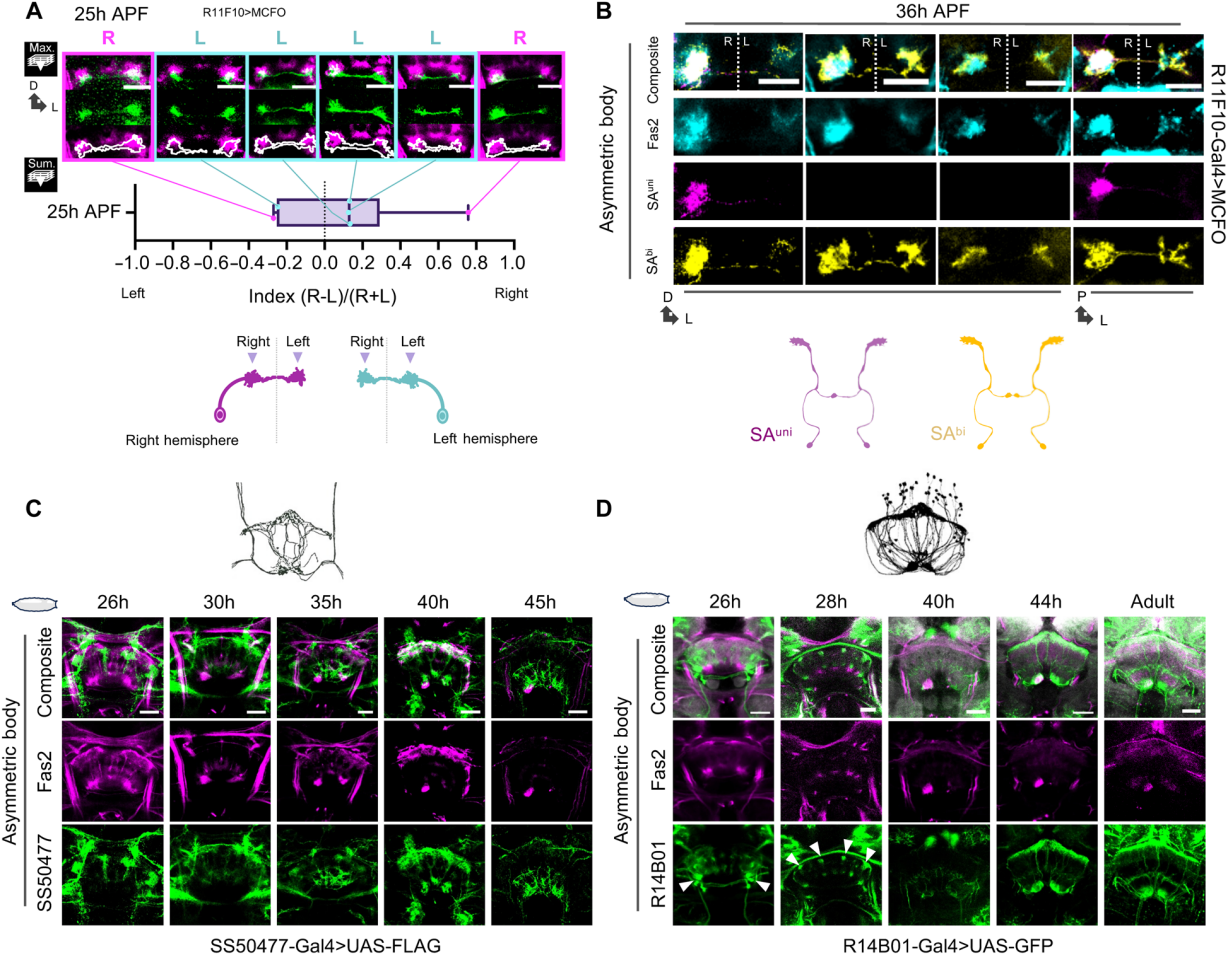
AB-FB relay connections develop after afferent remodeling. (**A** and **B**) Distinct developmental profiles of SA^uni^ and SA^bi^ axons during remodeling. (A) At 25 hours APF, individual SA^uni^ axons innervating the ventral AB primordium started to differ in density. Regardless of cell body position (R: right hemisphere; L: left hemisphere), neurons tended to innervate more densely in AB-R compared to AB-L. Immunofluorescence detection from MCFO (HA or FLAG) driven by *R11F10-Gal4* (green) and anti-Fas2 antibody staining (magenta). (B) Single-cell clones of SA^bi^ revealed their axonal class 1 asymmetry (yellow) next to SA^uni^ completely retracted from AB-L (purple; no SA^uni^ clones in columns 2 and 3). Immunofluorescence detection from MCFO (HA or FLAG) driven by *R11F10-Gal4* (magenta: clones segregated to the ventral primordium; yellow: clones in the dorsal primordium) and Fas2 antibody staining (cyan). (**C** and **D**) SA^FB^ afferents and v∆A efferent neurons project to the FB early but innervate AB-R and AB-L only after SA^uni^ and SA^bi^ remodeling is complete. (C) Immunodetection from *UAS-Flag* driven by *SS50477-Split-Gal4* (SA^FB^; green) and anti-Fas2 antibody staining (magenta). No SA^FB^ processes can be detected within AB-R (magenta) before 35 to 36 hours APF. (D) Dendrites of v∆A innervated the AB primordium earliest at 40 hours APF and showed different innervation patterns between AB-R and AB-L (45 hours APF). Immunodetection from *UAS-GFP* driven by *R14B01-Gal4* (green) and Fas2 antibody staining (magenta). All scale bars, 20 μm.

To match the Fas2-positive neurons in AB development with adult morphological types, we analyzed MCFO mosaics using *R72A10*-(SA^uni^), *R66A02*-(SA^bi^), and *R11F10-Gal4* (SA^uni^ and SA^bi^) lines (Fig. 5, A and B, and fig. S3; for details in the protocol of clonal and pupal developmental analyses, see Materials and Methods). While both SA^uni^ and SA^bi^ cell types formed bilateral axonal projections in early pupa stages, SA^uni^ neurons clustered to ventral AB-L and AB-R subregions (Fig. 5A), and SA^bi^ axons densely occupied the dorsal AB neuropils (Fig. 5B and fig. S3A). These dorsal and ventral axon domains merge during AB maturation, but SA^bi^ processes could still be found more densely packed dorsally in the adult AB-R (see Fig. 2D). With the described birth order–dependent dorsal-ventral sequence of FB layer innervation by tangential neurons (*17*), SA^bi^ neurons would be generated shortly before SA^uni^ as the last cell identity to emerge from the LALv1A hemilineage. In addition to the late-born SA^uni^ and SA^bi^ types, a third type of AB afferent neurons, SA^FB^ (*SS50477-SplitGal4*), emerges within the LALv1A hemilineage several divisions before SA^uni^ and SA^bi^ and projects toward the CX (*17*). In contrast to SA^uni^ and SA^bi^, SA^FB^ axons bypass the future AB primordium region and extend to the dorsal FB (Fig. 5C, 26 hours APF). Thus, the induction and asymmetric remodeling of the AB neuropil seem to be mediated by late-born SA^bi^ and SA^uni^ pioneer afferents with a similar bilateral projection pattern but distinct D/V domains of their axon terminals. At 25 hours APF, individual SA^uni^/SA^bi^ afferent neurons started to increase bilateral axon branching, which seems rather dynamic, and no clear lateralization could be recognized (Fig. 5A and fig. S3B). From 25 hours APF to the completion of lateralization at 36 hours APF, the SA^uni^ axonal processes underwent considerable remodeling, withdrawing from AB-L and restricting their innervation exclusively to AB-R (Fig. 5B). Axonal processes of single SA^uni^ neurons were not labeled by Fas2 antibody staining, suggesting that the onset of expression started during axonal remodeling or could be explained by strict localization of Fas2 protein within the AB neuropil (Fig. 5A). With the retraction of SA^uni^ axons from AB-L, the closely associated SA^bi^ axons also developed asymmetrically with denser innervations in AB-R, but sparse axonal processes remained at AB-L after the completion of SA^uni^ remodeling (Fig. 5B and fig. S3A). SA^bi^ neurons expressed Fas2 throughout developmental lateralization but gradually attenuated expression at 36 hours APF (Fig. 5B and fig. S3A). The loss of Fas2 expression in SA^bi^ at 40 hours APF indicated the completion of SA^uni^ and SA^bi^ axon remodeling (fig. S3A). In summary, remodeling SA^uni/bi^ axons show distinct Fas2 expression patterns with higher levels in the formation of SA^bi^ class 1 asymmetry compared to the class 2 asymmetry of SA^uni^.

Following AB primordium lateralization, SA^FB^ neurons, which first project to the dorsal FB, start to innervate the asymmetric AB neuropil in both hemispheres (Fig. 4C and fig. S4). During SA^uni/bi^ remodeling, SA^FB^ neuron processes accumulate in the ventral FB but remain outside of the AB neuropil (Fig. 5C and fig. S4). At 40 hours APF, first, SA^FB^ processes contact the AB neuropil and intermix with SA^uni/bi^ afferents in the following 10 hours with pronounced AB-R > AB-L asymmetry in fiber density (Fig. 5C and fig. S4). Like SA^FB^ afferents, no innervation of the bilateral AB neuropil by processes of v∆A neurons could be observed before 40 hours APF (Fig. 5D). v∆A neurons also targeted the dorsal and ventral FBs during early pupal development and formed restricted Fas2-negative fiber aggregates by 30 hours APF (Fig. 5D), reminiscent of early-born FB pontine neurons (*13*, *26*). In contrast to the symmetric growth pattern of SA^FB^ axons, extending dendritic processes of v∆A neurons differ between the left and right AB primordia, covering loosely the outer surface of AB-R while tightly converging in the center of AB-L (Fig. 5D, 40 hours APF). Notably, about 30% of v∆A neurons never invade the AB but retain the initial pontine projection pattern into the adult system, connecting FB layer 1 instead of AB to dorsal FB layers [Hemibrain data (*16*)]. In summary, AB circuit lateralization develops in a two-step process, in which unilateral afferent remodeling first built a neuropil primordium containing both class 1 (SA^bi^) and class 2 (SA^uni^) neuronal asymmetry, followed by bilateral innervation of FB neuron types (SA^FB^ and v∆A). Because of this sequential assembly, the loss of class 2 asymmetry in SA^uni^ observed in about 8% of *Drosophila* wild-type brains (*6*) would also affect the subsequent recruitment of FB neurons and the overall CX circuit lateralization.

### Dynamic Fas2 expression supports afferent lateralization independent of cell adhesion

**Fig. 6.**
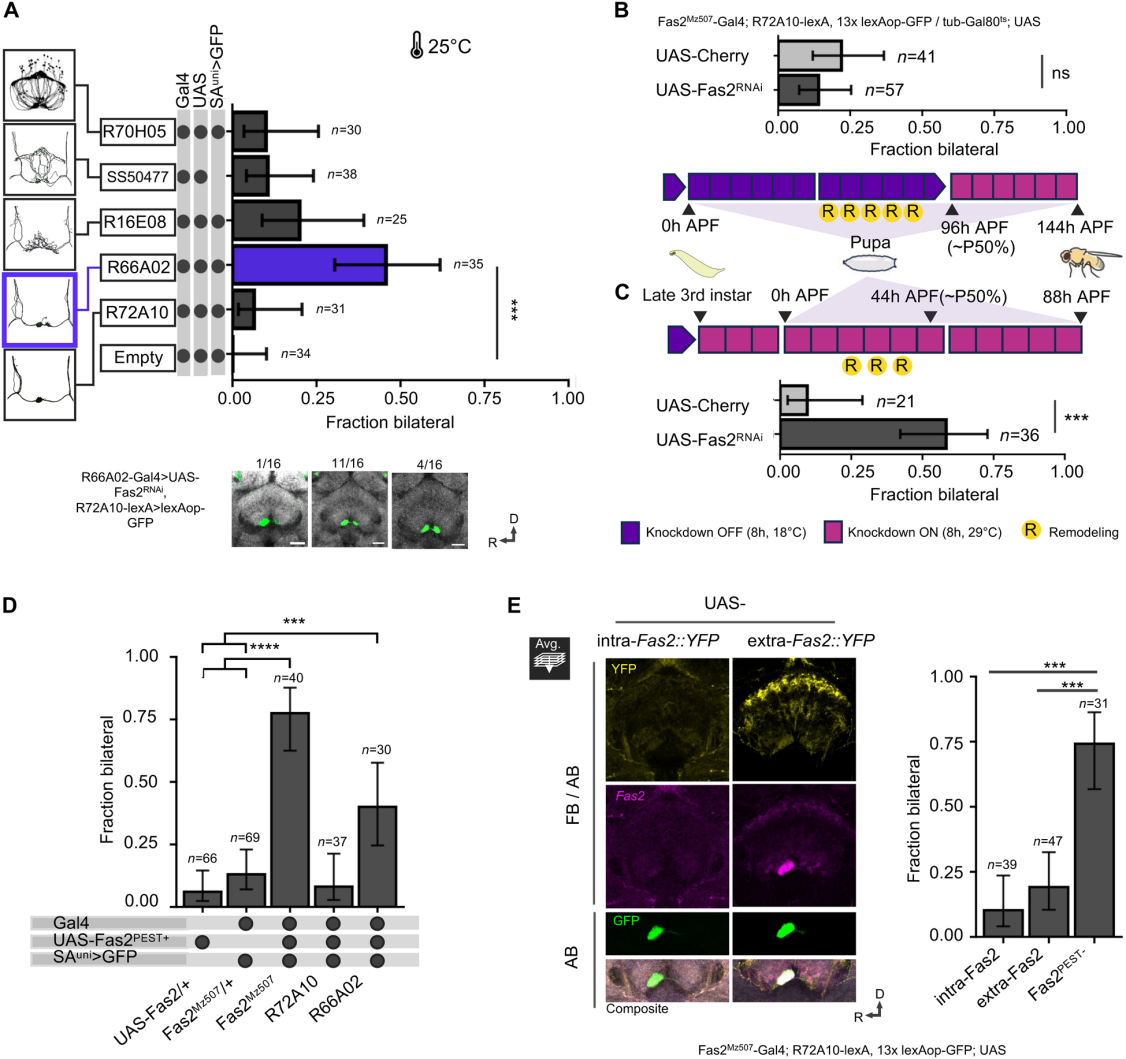
Fas2 function supports CX lateralization. (**A**) Fas2 knockdown by cell type–specific Gal4 lines for AB neurons showed that the loss of function in SA^bi^-specific *R66A02-Gal4* has a similar effect as loss in *Fas2^Mz507^-Gal4*–positive cells on the SA^uni^ projection pattern (χ^2^). (**B** and **C**) Temporally restricted developmental knockdown identified the critical period of Fas2 function in unilateral axon remodeling (R). Gal80^ts^-mediated suppression of targeted Fas2 knockdown (18°C; purple boxes) followed by the onset of targeted Fas2 knockdown at a restrictive temperature (29°C; red boxes). (B) Onset of Fas2 knockdown after SA^uni^ remodeling did not affect AB lateralization. (C) Onset of Fas2 knockdown with the transition from larva to pupa disrupted SA^uni^ remodeling. (**D**) Overexpression of the Fas2^PEST+^ isoform in SA^bi^ (*R66A02-Gal4* and *Fas2^Mz507^-Gal4*) increased the rate of brains with bilateral SA^uni^ projections (χ^2^). (**E**) Expression of *UAS-intra-Fas2^PEST-^::YFP* under the regulatory control of *Fas2^Mz507^-Gal4* resulted in a down-regulation of endogenous Fas2 (magenta) in the CX (AB-R, ventral and dorsal FB, and inner EB). Expression of *UAS-extra-Fas2^PEST-^::YFP* under the regulatory control of *Fas2^Mz507^-Gal4* results in ectopic expression of endogenous Fas2 in ventral and dorsal FB layers. No down-regulation of Fas2 expression was observed. Immunofluorescence detection of intra- or extra-*Fas2::YFP* driven by *Fas2^Mz507^-Gal4* (yellow), *lexAop-GFP* driven by *R72A10-lexA*, anti-Fas2 (D4) antibody staining (magenta), and anti-CadN antibody staining (gray). Truncated Fas2 isoforms under the control of *Fas2^Mz507^-Gal4* do not recapitulate the expressivity of full-length SA^uni^ projections. Error bars indicate two-sided 95% CIs for the single proportions. Significance thresholds are as follows: ***P < 0.001; ****P < 0.001.

With dynamic Fas2 expression in developing AB afferents and impaired lateralization following Fas2 reduction, we next used targeted RNAi knockdown to identify the neuronal identity of critical Fas2 expression (Fig. 6A). Given that the AB-R–specific innervation pattern of SA^uni^ neurons correlates with CX lateralization in the adult CX, we visualized their L-R innervation pattern (*R7210-lexA*, *LexOpGFP*) following the targeted manipulation of Fas2 expression. Developmental loss of Fas2 in SA^uni^ did not affect unilateral AB-R innervation (Fig. 6A), confirming results in (*15*). In contrast, knockdown of Fas2 in SA^bi^ (*R66A02-Gal4*) was able to replicate the effect of the manipulation under the control of *Fas2^Mz507^-Gal4* (Fig. 6A compared to Fig. 4B). As indicated by single-cell clones, SA^bi^ axons segregate into the dorsal AB subregion, which correlates with the *Fas2^Mz507^-Gal4* expression (Fig. 4G), but only transiently express Fas2 protein (Fig. 5B and fig. S3A). Different developmental windows of targeted Fas2 knockdown via Gal80^ts^ regulation (*27*) confirmed the functional requirement of Fas2 during AB remodeling in early pupae (Fig. 6, B and C). While a Fas2 knockdown starting at 50% pupal development (shortly after AB remodeling) does not affect SA^uni^ connectivity (Fig. 6B), the onset of Fas2 RNAi during larva/pupa transition is sufficient to block AB lateralization (Fig. 6C; see Materials and Methods for experimental details).

To further investigate a potential role of Fas2-mediated cell adhesion in CX lateralization, we expressed different Fas2 isoforms (*UAS-Fas2^PEST+^ and UAS-Fas2^PEST−^*) in AB afferent neurons (Fig. 6, D and E). Overexpression of transmembrane isoforms of Fas2 in SA^bi^ axons (*Fas2^Mz507^-Gal4* and *R66A02-Gal4*) strongly impairs asymmetric remodeling of SA^uni^ processes (~75% of brains showing bilateral innervation of SA^uni^ neurons; Fig. 6D). In contrast, overexpression of just the intracellular or extracellular domains of Fas2 [*UAS-intra-Fas2^PEST-^::YFP* and *UAS-extra-Fas2^PEST-^::YFP* (*22*)] under the control of *Fas2^Mz507^-Gal4* did not affect lateralization (Fig. 6E). The overexpression of Fas2 in SA^uni^ (*R72A10-Gal4*) had no effect on the differentiation of SA^uni-L^ and SA^uni-R^ (Fig. 6D). Together with the SA^bi^-specific loss-of-function phenotype, these data indicate a nonautonomous function of Fas2 in SA^uni^ remodeling, independent of Fas2-mediated SA^uni^-SA^bi^ adhesion. Furthermore, the extracellular domain alone could not recapitulate the overexpression effect that we observed with the full transmembrane isoforms (Fig. 6E). The intracellular domain of Fas2 was shown to be involved in neuronal remodeling processes (*24*) and synaptic growth (*28*). As Fas2 can also act as a non–cell-autonomous repressor of endothelial growth factor receptor (EGFR) signaling (*29*) and EGFR itself controls synaptic stabilization (*30*), we modified the EGFR function during AB development. However, no changes in SA^uni^ remodeling following the expression of a constitutively active form (*UAS-EGFR*^λ*TOP*^) or a dominant-negative mutant (*UAS-EGFR^DN^*) could be observed (fig. S6). To test whether the Fas2 function is restricted to the interactions between SA^uni^ -SA^bi^ afferents, we expressed Fas2^RNAi^ in the FB neurons v∆A (*R70H05-Gal4*) and SA^FB^ (*SS50477*). Knockdown of Fas2 in v∆A and SA^FB^ had no effect on AB lateralization (Fig. 6A), consistent with our observation that these cell types innervate the AB after SA^uni^/SA^bi^ remodeling. These results reveal a critical role of SA^uni^-SA^bi^ cellular interaction in CX lateralization, in which the bilateral SA^bi^ supports the class 2 asymmetry of SA^uni^ innervation.

### Bilateral SA^bi^ neurons organize structural and functional CX lateralization

**Fig. 7.**
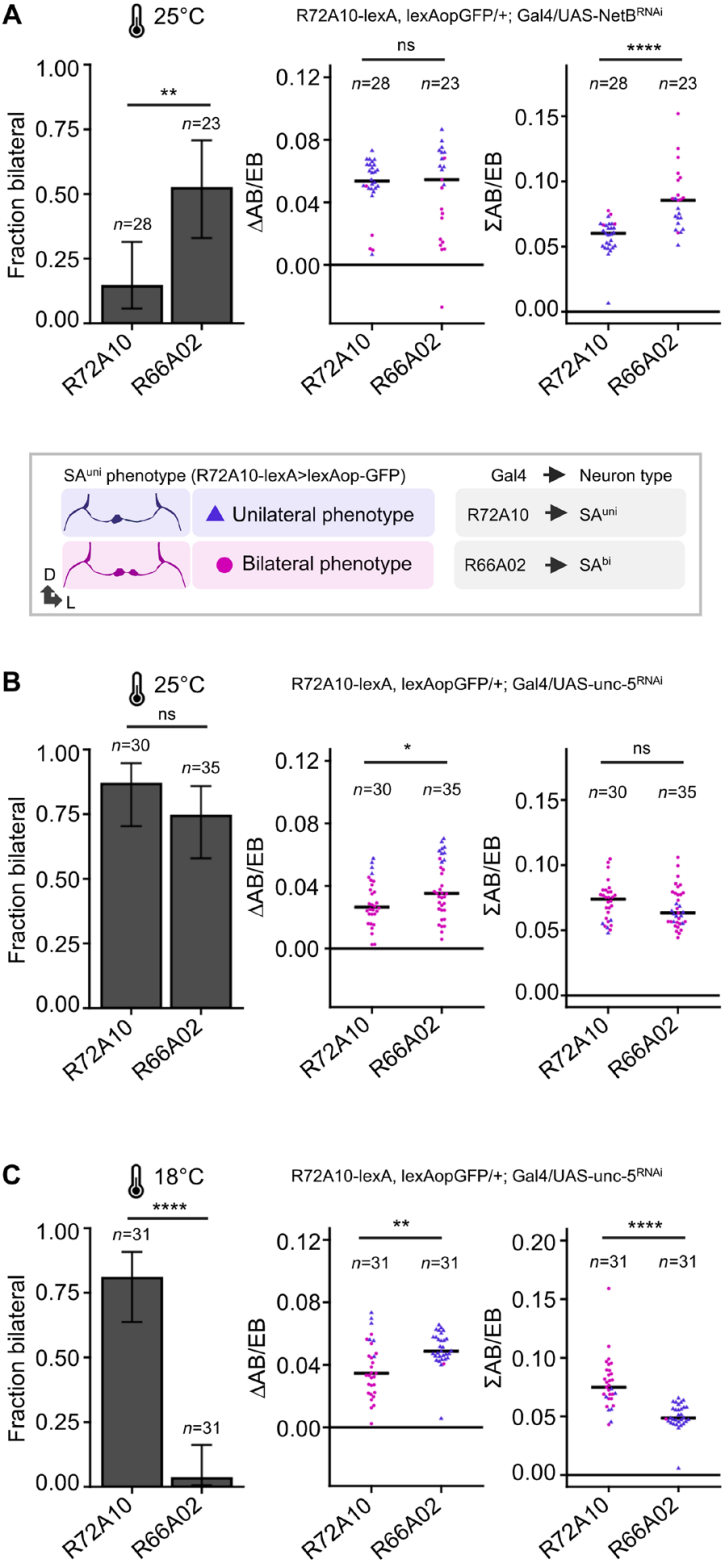
Netrin signaling is differently required by SA^uni^ and SA^bi^. (**A**) At the standard developmental temperature, the rate of brains with SA^uni^ projecting bilaterally to AB-R and AB-L is statistically significantly higher when NetB^RNAi^ is expressed in SA^bi^ compared to expression in SA^uni^ (χ^2^). While the effect on input lateralization (∆AB/EB) is low and not statistically significantly different between NetB^RNAi^ expressed in SA^uni^ and SA^bi^, SA^uni^ has a larger AB innervation area (∑AB/EB) following the loss of *NetB* in SA^bi^ (Mann-Whitney *U*). (**B**) Loss of unc-5 receptor expression in SA^bi^ causes non–cell-autonomously a comparable rate of bilaterally projecting SA^uni^ as targeted *UAS-unc-5^RNAi^* in SA^uni^ (χ^2^), although the effect on SA^uni^ lateralization (∆AB/EB) is weaker (Mann-Whitney *U*). (**C**) In contrast to Fas2 manipulation, developmental temperatures showed different sensitivity of targeted *unc-5* knockdown in SA^uni^ and SA^bi^. While there is no obvious difference in the cell-autonomous effect on SA^uni^ morphology (Mann-Whitney *U*), the non–cell-autonomous effect of unc-5 knockdown in SA^bi^ was no longer detectable (χ^2^). The innervation area was measured from SA^uni^ labeling in AB-R and AB-L. Error bars indicate two-sided 95% CIs for the single proportions. Crossbars in area measurements indicate group medians. Blue triangles indicate measurements from brains with a unilateral AB projection by SA^uni^, and magenta dots indicate brains with a bilateral projection. Significance thresholds are as follows: *P < 0.05; **P < 0.01; ****P < 0.001.

**Fig. 8.**
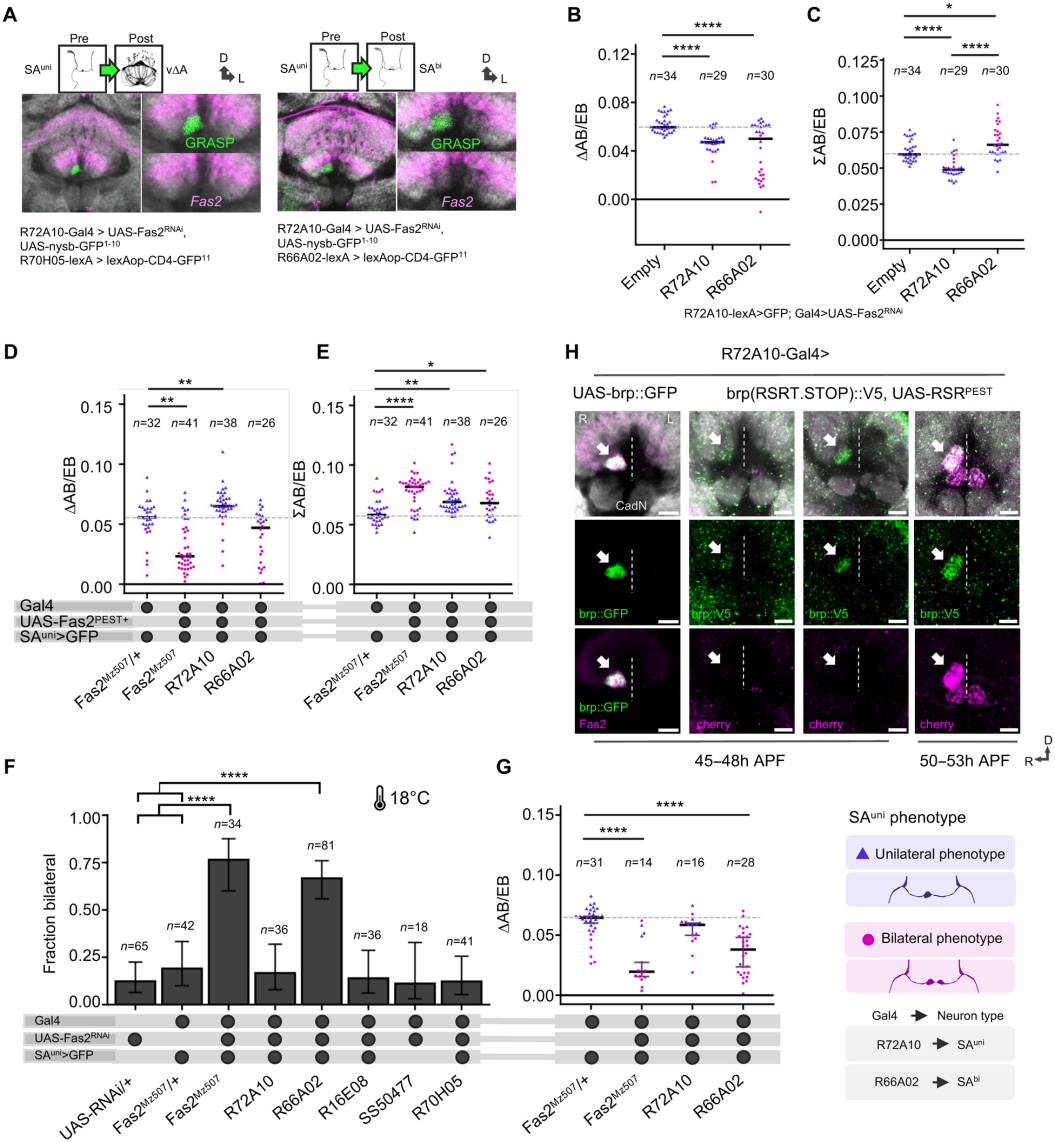
Differential Fas2 expression delayed afferent-afferent synapse formation. (**A**) Immunofluorescence detection of *nsybGRASP* (green) showed that SA^uni^ formed and maintained functional synapses in the absence of cell-autonomous Fas2 expression. Anit-Fas2 (magenta) and anti-CadN (gray) antibody staining. (**B**) Loss of Fas2 reduced SA^uni^ lateralization cell-autonomously (*R72A10-Gal4*) and non–cell-autonomously (*R66A02-Gal4*) (Kruskal-Wallis/Dunn). (**C**) The bilateral brains had greater total innervation area, resulting in a greater area for the *R66A02-Gal4* experimental group. Loss of Fas2 in SA^uni^ reduced the innervation area without affecting the projection pattern (Kruskal-Wallis/Dunn). (**D**) Overexpression of the Fas2^PEST+^ isoform in drivers that label SA^bi^ (*R66A02-Gal4* and *Fas2^Mz507^-Gal4*) and decreased innervation area lateralization (Kruskall-Wallis/Dunn). (**E**) The normalized AB innervation area of SA^uni^ increased in all Fas2 overexpression experiments (Kruskall-Wallis/Dunn). (**F**) Targeted RNAi knockdown of Fas2 at 18°C developmental temperature increased the expressivity of the bilateral SA^uni^ phenotype (χ^2^) and (**G**) reduced lateralization when driven by *Fas2^Mz507^-Gal4* or *R66A02-Gal4* (Kruskal-Wallis/Dunn) despite lower Gal4/UAS efficiency compared to 25°C. (**H**) Immunodetection of brp::GFP (green), under the control of *R72A10-Gal4* (SA^uni^), was used as an axonal marker and was detectable in SA^uni^ AB innervations before the expression of the endogenous brp (tagged with V5 upon *R72A10-Gal4*–driven excision of an *RSRT-STOP* codon), the onset of which started at 48 hours APF. This indicates that chemical synapses do not become functional until after afferent remodeling. Blue triangles indicate measurements from brains with a unilateral AB projection by SA^uni^, and magenta dots indicate brains with a bilateral projection. Crossbars indicate medians. Error bars indicate two-sided 95% CIs for the single proportions. Significance thresholds are as follows: *P < 0.05; **P < 0.01; ****P < 0.001.

To further characterize the role of SA^bi^ neurons in supporting SA^uni^ lateralization, we analyzed the previously described axon guidance molecule Netrin-B (NetB), which signals repulsion via the unc-5 receptor, but for which the cellular origin has not been identified (*9*). Targeted knockdown of NetB in SA^bi^ driven by *R66A02-Gal4* and *Fas2^Mz507^-Gal4* resulted in a strong reduction of SA^uni^ remodeling compared to the control, while knockdown in developing SA^uni^ neurons (*R72A10-Gal4*) had no effect on AB lateralization (Fig. 7A). Targeted knockdown of unc-5 in developing SA^bi^ also affects SA^uni^ remodeling (Fig. 7B), indicating that the combined initial retraction of both afferent types from AB-L (as described above) is critical for the morphological transition of class 1 to class 2 asymmetry of SA^uni^ neurons (Fig. 4A) (*9*). This differential sensitivity to repulsive Netrin signaling is further supported by the loss of a remodeling phenotype for SA^uni^ following a temperature-mediated reduction in the level of unc-5 knockdown (Fig. 7C). Although the total number of flies with bilateral SA^uni^ innervation (penetrance) is higher in samples with SA^bi^-targeted NetB knockdown, the reduction of interhemispheric asymmetry (expressivity) in experimental brain with bilateral SA^uni^ innervation is stronger following the loss of Fas2 function in SA^bi^ (Figs. 7A and 8B) {∆AB/EB, *R66A02* versus empty-Gal4; rank biserial: *NetB*^*RNAi*^: *r* = 0.04 [95% confidence interval (CI): −0.23, 0.30]; tiny effect versus *Fas2*^*RNAi*^: *r* = 0.29 [95% CI: 0.05, 0.49], indicating a medium effect}. Together, directional signaling between AB pioneer afferents diversifies their interhemispheric connectivity. Although unilateral axon remodeling is initiated in both SA^uni^ and SA^bi^ neurons, the complete transition from class 1 to class 2 asymmetry of SA^uni^ requires Fas2 function and NetB signaling in SA^bi^.

As AB circuitry has been described to be involved in olfactory learning (*31*) and satiety-dependent sugar preference (*32*), with lateralization of SA^uni^ in particular being critical for aversive olfactory and courtship long-term memory (*7*, *9*), we wanted to test whether manipulation of bilateral SA^bi^ neurons is sufficient for memory impairment. We found that 24-hour memory expression in flies trained in a single-session appetitive olfactory conditioning paradigm (*33*) was significantly reduced following Fas2 overexpression in SA^bi^ (*UAS-Fas2^PEST+^* under the control of *R66A02-Gal4*; fig. S5, A and C). Similarly, postfasting fructose preference was lost in flies with SA^bi^-induced reduction in AB lateralization (fig. S5B), supporting the importance of developmental AB afferent interactions for efficient information processing in the mature brain (see Materials and Methods for experimental details).

### Fas2 delays afferent-afferent synapse formation

As the loss of Fas2 in developing SA^uni^ allows its axons still to aggregate at the positions comparable to the wild-type AB and to segregate from the adjacent CX neuropils (Fig. 4E), the Fas2 function is not necessary for afferent axon guidance and bilateral AB neuropil formation. Using the nSyb-GRASP construct, we were able to confirm the presence of functional synapses in the adult AB circuit even in the absence of Fas2 (Fig. 8A), suggesting that connections were not eliminated as described for synapses in the neuromuscular junction (*23*).

As Fas2 has been described to modulate the number of presynaptic sites depending on the expression level (*23*), we next examined a potential role of Fas2 in synaptic growth within the AB circuit. We measured the area of SA^uni^ axonal innervations in the ABs following Fas2 knockdown (Fig. 8, B and C) or overexpression (Fig. 8, D and E). When under the control of *R72A10-Gal4*, the normalized area of AB innervations by SA^uni^ was decreased under the Fas2 knockdown condition and increased by Fas2 overexpression, thus affecting CX input lateralization without altering class 2 asymmetry projection. This supports a cell-autonomous function of Fas2 in SA^uni^ lateralization at the level of synaptic growth. While bilateral projections of SA^uni^ typically lead to smaller innervations in AB-R, the sum of the innervation cross-sectional areas in AB-R and AB-L in the bilateral phenotype (both in wild-type variations and in experimental groups) tends to be larger than the area in the unilateral AB-R morphology (Figs. 4, C and D; 7; and 8, B to E), indicating additional ectopic synapses of bilateral SA^uni^ projections.

A previous study has shown that reduced developmental temperature results in an increased formation of ectopic synapses during *Drosophila* pupal stages (*34*).To test whether the loss of Fas2 expression in SA^bi^ supports the formation of ectopic synaptic connections of SA^uni^, we prolonged AB remodeling via changes in environmental temperature (Fig. 8F). We compared the SA^uni^ asymmetry pattern as the normalized difference between AB-R and AB-L following the targeted Fas2 knockdown in SA^bi^ at 18° and 25°C. Here, the expressivity in the reduction of SA^uni^ asymmetry was higher at a developmental temperature of 18°C compared to 25°C (Fig. 8G). Despite a reduced RNAi efficiency at lower temperatures, the effect increased significantly at 18°C compared to 25°C (rank biserial, *r* = 0.84 [95% CI: 0.69, 0.92], large effect at 18°C; *r* = 0.36 [95% CI: 0.08, 0.59], indicating a large effect at 25°C). These results support a model in which different levels of Fas2 expression between closely associated axon branches of SA^uni^ and SA^bi^ prevent interaxonal synapse formation before the remodeling of AB pioneer afferents. In line with a delayed synapse formation via differential Fas2 expression dynamics, first signs of presynaptic differentiation of SA^uni^ neurons could be observed after AB remodeling [synaptic tagging with recombination (*35*)] (Fig. 8H). In summary, dynamic Fas2 expression of AB pioneer afferents organizes two aspects of CX lateralization: diversification of lineage identities in unilateral axon remodeling and functional circuit lateralization during synaptogenesis.

## DISCUSSION

Despite its omnipresence in nervous systems throughout the animal kingdom to support cognitive performance and behavioral plasticity, little is known about how brain lateralization is organized and established within neural circuits. As structural and functional differences of brain circuits between the right and left hemispheres are deeply embedded in an overall bilaterally symmetric nervous system, understanding the developmental programs that induce local symmetry breaks without interfering with symmetric pattern formation will provide important insights into the function and dysfunction of brain laterality. Using the *Drosophila* CX as a conserved brain structure in which a multitude of tangential and columnar neuron types integrates in a highly symmetric bilateral neuropil, we show how the interaction of two bilateral afferent neurons creates local asymmetry followed by the synaptic integration and secondary lateralization of default symmetric CX neurons.

**Fig. 9.**
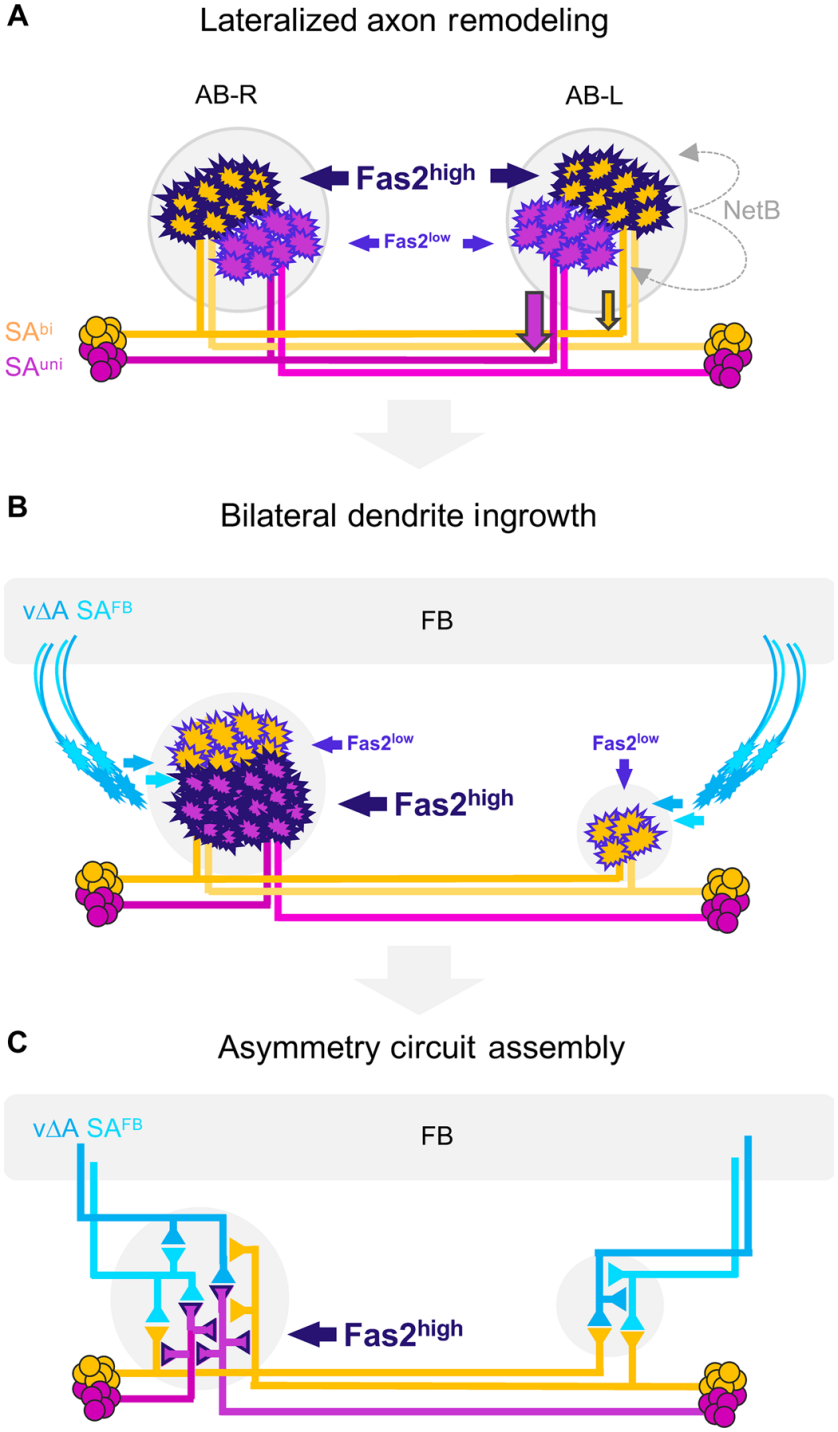
Sequential cell-cell interactions built CX circuit asymmetry in *Drosophila*. (**A**) Interactions between two types of AB pioneer neurons initiate lateralized axon remodeling. Different levels of Fas2 expression in SA^uni^ (low) versus SA^bi^ (high) support class-specific, NetB-mediated axon retraction (partial or complete for SA^bi^ or SA^uni^, respectively). (**B**) Following the completion of AB afferent lateralization, SA^uni^ and SA^bi^ switch their relative levels of Fas2 expression to high or low, respectively, accompanied by the bilateral dendrite ingrowth of FB relay neurons v∆A and SA^FB^. (**C**) The unilateral localization of Fas2-positive SA^uni^ axon terminals results in side-specific AB microcircuits, with dominant afferent-afferent connectivity in AB-R versus afferent-efferent connectivity in AB-L.

Lateralization of the CX depends on the interhemispheric interaction of two types of AB pioneer neurons, SA^uni^ and SA^bi^, which not only initiate the formation of the AB primordia but also regulate the remodeling of a bilaterally symmetric ground state. Following unilateral retraction of SA^uni^ axons, earlier-born FB neurons are recruited into the AB primordium, including both SA^FB^ tangential neurons of the same lineage but also columnar v∆ neurons of the conserved DM lineages. SA^bi^ neurons instruct SA^uni^ lateralization via the cell surface molecule Fascilin 2 and the secreted guidance factor Netrin-B. Although both AB afferent neurons derive from the same temporal window of the vLAL1A lineage, the SA^uni^ cell fate is specified in the last 8 to 10 final NB divisions, which might result in distinct differentiation states of the two afferent neurons by the onset of axon remodeling. While both cell types start to retract their axons from AB-L, SA^bi^ neurons maintain some processes in the bilateral state and therefore differ from the unilateral innervation of their sister neurons. From this developmental perspective, the bilateral morphotype of SA^uni^ can be explained by the incomplete segregation from their SA^bi^ sister neurons (Fig. 9).

Several findings indicate an active role of SA^uni^ in synaptic recruitment. First, lineage-related SA^bi^ and SA^FB^ form a large number of synapses with SA^uni^ in AB-R, whereas such afferent-afferent connections are almost absent in AB-L. Second, during development, v∆A first forms denser innervations in AB-L before invading the space occupied by Fas2-positive SA^uni^ in AB-R. This L-R difference is lost in brains with bilateral SA^uni^ innervation. Third, lineage-related LCNOpm neurons stochastically extend branches toward AB-R and, in rare cases, form a substantial number of synapses, as in the brain imaged for the Hemibrain dataset (*16*). Both axonal prepatterning of the AB primordia and secondary FB dendrite recruitment reveal an unexpected amount of structural plasticity in conserved CX circuitry up to mid-pupal development. While fine-tuning of branching and synaptic growth also occurs in the second half of pupal development (*30*, *34*), the pattern of neuropil connectivity for bilaterally symmetric central brain circuits has already been finalized [with the exception of adult circadian plasticity (*36*)]. As physical proximity in the transition from growth to connectivity is a strong predictor of synaptic partner choice (*37*), a delay in the retraction from AB-L would allow SA^uni^ axons to recognize SA^bi^ processes as synaptic partners and therefore prevent their lateralized retraction.

Neuronal remodeling of major neuronal circuits in *D. melanogaster* occurs in a synchronized manner in the central and peripheral nervous systems and is regulated by ecdysone signaling [reviewed in (*38*)]. The initial pruning is completed by 20 hours APF (*38*), i.e., before the AB primordium is formed. While ecdysone signaling was shown to be involved in establishing the left-right axis in the brain, SA^uni^ remodeling is initiated independently of ecdysone as lateralization is unaffected by the expression of a dominant-negative allele of the receptor EcR in AB neurons (*15*). In close proximity to the developing AB neuropil, FB neurons establish a commissural primordium in first instar larva consisting of undifferentiated neurites from embryonal cells (*13*). The later ingrowth of secondary axons from later-born neurons results in the maturation to the adult neuropil (*13*). This sequence is reversed in AB development, where late-born tangential neurons form the primordium and establish the lateralized blueprint in early pupal development before early-born columnar neurons are recruited secondarily. During CX development, projections of columnar neurons establish arborizations in FB, PB, EB, and NO by 28 hours APF (*39*). Pontine neurons form arborizations in the ventral FB later but are established by 40 hours APF (*39*), a time point when v∆A starts invading the AB. Considering that roughly one-third of v∆A neurons develop into pontine neurons and do not extend dendritic branches into the AB neuropils, the columnar v∆A projection pattern could have evolved from modified pontine neurons, which gained responsiveness to AB-derived signals. Delayed neuronal differentiation and synaptogenesis have been identified as key features of human cortex development supporting the generation of species-specific cell types and circuit motifs [reviewed in (*40*, *41*)]. In the *Drosophila* CX, the maintained responsiveness of intrinsically left-right symmetric neurons to guidance cues enables a small number of neurons to introduce a substantial level of asymmetry into the circuit. So far, the AB has only been described as a separate neuropil in *Diptera* (*42*). Physical segregation from the ventral FB might facilitate the asymmetrical prepatterning without affecting the broader CX organization.

We found the *Drosophila* neural cell adhesion molecule homolog Fas2 to support both the asymmetric prepatterning of the AB neuropils and the synaptic integration of the mature AB circuitry. Cell adhesion molecules are critical factors during circuit assembly and remodeling, specifically their spatiotemporal control (*43*). AB neuropils and afferent synapses do form in the absence of Fas2, indicating that the Fas2 function in axon guidance (*23*, *44*) and synaptic elimination (*23*) is not critical. In line with the involvement of Fas2 in synapse formation, growth, and stabilization (*23*, *45*, *46*), we found support for a traditional role of Fas2 in SA^uni^ promoting synaptic growth indirectly measured by the AB innervation area. However, we did not detect any cell-intrinsic effect of the Fas2 expression level in SA^uni^ on its neurite lateralization. A simple model in which Fas2-mediated trans-adhesion must be down-regulated in AB-L to allow retraction of SA^uni^ axons, analogous to the remodeling of the mushroom body γ-lobe in which Fas2 must be down-regulated to allow infiltration of astrocyte-like glia for pruning (*24*, *47*), finds no support in our data. On the contrary, our data are most consistent with the assumption that during remodeling of SA^uni^, Fas2 functions non–cell-autonomously and nonadhesively to promote afferent lateralization. In SA^bi^ neurons, Fas2 appears to function as a signaling molecule. This is supported by the finding that both extracellular and intracellular domains are required to recapitulate the reduction in AB lateralization observed in the Fas2^PEST+^ overexpression experiments.

A strong correlation between brain lateralization and cognitive performance has been recognized in humans and other animals. How the interplay between genetic programs and environmental factors influences adult brain lateralization is poorly understood. A reduction in the bilateral asymmetry of SA^uni^ neurons has been shown to affect different aspects of *Drosophila* memory formation. Here, we show that the genetic manipulation of a bilateral sister neuron of SA^uni^ results in cognitive performance in the context of appetitive olfactory memory and feeding-related decision-making. This further increases the already substantial number of affected cognitive functions (*7*, *9*, *32*, *48*). The degree of lateralization is affected by environmental factors. The dynamic transient growth state before and during remodeling appears to be sensitive to developmental temperature. A low temperature led to lower lateralization in general by increasing the frequency of the fluctuating symmetrical SA^uni^ phenotype. We interpreted this as an effect of the increase in ectopic synaptic connections for extended developmental time (*34*) and as evidence that neuronal proximity determines asymmetric synaptogenesis. However, it has been shown that the higher number of synaptic connections established at low developmental temperatures may be adaptive (*34*).

In summary, our results demonstrate that CX lateralization initially arises through a robust genetic program, involving two types of sequentially born pioneer neurons, which segregate their axons from a commissural system via interhemispheric cellular interactions. The nonadhesive and non–cell-autonomous function of Fas2 supports lateralized remodeling, a process that is notably sensitive to environmental factors. The secondary recruitment of processes from bilaterally symmetric CX neurons is intrinsically more variable and depends on the initial degree of afferent asymmetry as well as more dynamic cell-cell interactions, thereby resulting in interindividual variations.

Although we provide strong evidence for the importance of bilateral afferent interaction in circuit lateralization including the “split-brain” experiments, the identity of the initial symmetry breaking mechanism is still unknown. Additional approaches are required to distinguish between intrinsic L-R differences of AB afferent neuron clusters and an asymmetry signaling pathway in the cellular neighborhood of the AB neuropil.

Furthermore, the described developmental sequence of AB circuit assembly in which initially symmetric neurons become integrated into the lateralized precursor neuropil offers a plausible mechanistic explanation for the cell type–specific level of synaptic asymmetry described by the connectome. However, out experimental data are derived from confocal microscopy combined with transgenic transsynaptic labeling. Future studies would need to compare the developmental profile of synaptogenesis at EM resolution between wild-type and mutant lines with reduced AB asymmetry.

## MATERIALS AND METHODS

### *Drosophila* strains and genetics

All *D. melanogaster* strains were reared on standard medium and maintained at 25°C, unless otherwise stated in Materials and Methods. For immunohistochemical analysis of adult brains, flies of both sexes were selected for dissection 3 to 5 days after hatching. Conditioning was performed on 4- to 9-day-old mixed-sex flies. Saturation-dependent fructose drive was analyzed in 5- to 9-day-old mated females. Transgenic constructs were expressed under the control of previously described GAL4 lines: R72A10-Gal4 [Bloomington Drosophila Stock Center (BDSC), no. 48306], R72A10-lexA (BDSC, no. 54191) (*49*), R52H03-Gal4 (BDSC, no. 38849) (*50*), R11F10-Gal4 (BDSC, no longer available) (*50*), R66A02-Gal4 (BDSC, no. 39384) (*50*), R66A02-lexA (BDSC, no. 52688), R16E08-Gal4 (BDSC, no. 39416) (*50*), R70H05-Gal4 (BDSC, no. 39554) (*50*), R38D01-Gal4 (no. 49996) (*50*), R14B01-Gal4 (BDSC, no. 48597) (*50*), R14B01-lexA (BDSC, no. 52467) (*49*), SS50477 (BDSC, no longer available) (*11*), Fas2-Gal4^Mz507^-Gal4 (B. Altenhein), and Fas2^MiMIC12989^-Gal4 (BDSC, no. 77831) (*51*). For neuronal labeling and clonal analysis, we used MCFO (MCFO, BDSC, no. 64086) (*52*), Flybow 1.1B (BDSC, no. 56802) (*53*), 13x-lexAop-mCD8::GFP (Chr.II) (unknown source), 13x-lexAop-mCD8::GFP (Chr.III, BDSC, no. 32203), 10x-UAS-mCD8::GFP (BDSC, no. 32186), 10xUAS-mCD8::RFP & 13x-lexAop-mCD8::GFP (BDSC, no. 32229), UAS-mCD8::cherry (BDSC, no. 27392), and 10xUAS-FLAG (BDSC, no. 62147). For experimental manipulation of Fas2 expression, we used the following: UAS-Fas2^RNAi^ (BDSC, no. 28990) (*54*), UAS-Fas2^PEST+^ (*55*), UAS-Fas2^PEST-^ (*55*), UAS-intra-Fas2^PEST-^::YFP (A. Nose) (*22*), and UAS-extra-Fas2^PEST-^::YFP (A. Nose) (*22*). We manipulated EGFR signaling with UAS-EGFR^λTOP^ (BDSC, no. 59843) (*56*) and UAS-EGFR^DN^ (BDSC, no. 5364) (*57*). For the temporal control of Fas2 knockdown, we used tub-Gal80^ts^ (BDSC, no. 7108) (*27*) and UAS-Cherry-(VALIUM10) (BDSC, no. 35787) (*54*) as the control group for RNAi expression. We used UAS-unc-5^RNAi^ (BDSC, no. 33756) (*54*) and UAS-NetB^RNAi^ (BDSC, no. 25861) (*54*) for loss-of-function experiments. We performed induced apoptosis with UAS-hid^14^ (J. R. Nambu). We used Nrg^849^ (BDSC, no. 35827) to prevent the development of interhemispheric commissures. We used the following constructs to analyze synaptic configuration and connectivity: UAS-syt::GFP (BDSC, no. 6925), UAS-DenMark::cherry (BDSC, no. 33062) (*58*), UAS-brp::GFP (BDSC, no. 36291), trans-Tango (BDSC, no. 77124) (*59*), nysb-GRASP (BDSC, no. 64315) (*60*), t-GRASP (BDSC, no. 79039) (*61*), brp(RSRT.Stop)V5-2A-LexA (*35*), and 20xUAS-RSR^PEST^ (BDSC, no. 55756) (*35*). Documentation of all fly strains with research resource identifiers (table S1) and of all crosses and genotypes (data S1) can be found in the Supplementary Materials.

### Fly injections for empty-Gal4

A GC 100-10 glass capillary was prepared with a needle puller, and the tip of the capillary was ground at an angle of 12° for ~30 s using a capillary grinder. The capillary was then connected to a 10-ml injection syringe and rinsed several times with acetone.

Embryos of the nanos-phiC31;;attP2 genotype (BDSC, no. 99002) were prepared for the injections by rinsing them in chlorine solution (1:1 DanKlorix and tap water) and arranging them in rows on coverslips coated with cello adhesive and submerged in H10S oil (Arkema). Empty plasmid pBPGUw (Addgene) was extracted from bacterial strain DB3.1 and injected into the embryos in buffer solution [0.77 ml Na_2_HPO_4_ (1 M), 0.22 ml NaH_2_PO_4_ (1 M), 3.728 g KCl, and fill up with water to a total volume of 1 liter] and phenol red. Injected embryos were immersed in H3S oil (Arkema). Hatching larva was collected and transferred to standard food medium. All the resources used are documented in table S1.

### Nomenclature

In the Hemibrain dataset, the SLP-AB (*11*) category is divided into three neuronal categories: SA1, SA2, and SA3. We wanted to stick as closely as possible to the Hemibrain nomenclature but emphasized that SA1 and SA2 are the corresponding right and left hemispheric morphs of the same cell type, whereas SA3 is a separate type. Therefore, we have summarized SA1 and SA2 as SA^uni^ (after their typical unilateral AB-R projections) and renamed SA3 to SA^bi^ (for their bilateral projection pattern to AB-R and AB-L). SA^uni^ has also been referred to as Janus neurons (*32*) and H neurons (*9*) in publications. We have also named SAF SA^FB^ in this study to emphasize its similarity to the other afferents but to refer to its specific projection to the dorsal FB.

### Connectome analysis

We accessed the Hemibrain EM dataset (version 1.2.1) (*16*) for connectome analysis via the neuPrint+ (*18*) browser interface (https://neuprint.janelia.org). neuPrint+ output from custom queries was downloaded and processed in R (*62*) [version 4.4.1; tidyverse version 2.0.0 (*63*)].

### RNAi and overexpression experiments

Upstream activator sequence (UAS)–construct flies were crossed with Gal4 driver lines, and crosses were kept at 25° or 18°C. As a control for Rubin lab Gal4 lines (*50*), we crossed empty-Gal4 with the same UAS construct to maintain the genetic background in the controls, whereas in experiments with Fas2^Mz507^-Gal4, we crossed both Gal4 and UAS with Canton S. We evaluated the SA^uni^ projection pattern using immunodetection of *R72A10-lexA* driving *lexAop-mCD8::GFP*.

### Temporal control of RNAi expression

The Fas2^Mz507^-Gal4 construct was used to express the UAS-Fas2^RNAi^ transgene. The temperature-sensitive tub-Gal80ts allele (*27*) restricts Gal4/UAS expression at 18°C, whereas it is permissive to Gal4/UAS expression at 29°C. Crosses were kept at 18°C.

To test the effect of Fas2 knockdown after afferent remodeling is complete, pupae were collected within the first 30 min after pupation (P0). After 96 hours, the pupae were transferred to 29°C to activate UAS-RNAi expression. Ninety-six hours APF was chosen because pupal development takes 2.05 times longer at 18°C than at 25°C (*34*), where we found afferent remodeling to be complete after 40 hours APF.

To test the requirement of Fas2 expression during remodeling, we collected late third instar pupae and immediately transferred them to 29°C to activate UAS-Fas2^RNAi^ expression. Given that temperature may influence physiological processes, a control group expressing UAS-cherry-(VALIUM10) instead of UAS-Fas2^RNAi^ underwent the same temperature regime as the experimental group.

Flies with the correct genotype were dissected 3 to 5 days after eclosion. We evaluated the SA^uni^ projection pattern using immunodetection of *R72A10-LexA* driving *LexAop-mCD8::GFP*.

### Phenotype classification

The projection phenotypes were divided into two categories: unilateral (innervations only detectable in AB-R) and bilateral (innervations in AB-R and AB-L). If the row and column totals were greater than 5, a chi-square test was performed. Otherwise, the Fisher exact test was used to test for statistically significant effects. We corrected for multiple comparisons using the Bonferroni method. We defined phenotypic penetrance as the rate of bilateral phenotypes; that is to say, it is the ratio of brains exhibiting bilateral SA^uni^ innervation to the total number of brains of a given genotype. All statistics were performed in R (version 4.4.1) (*62*). Plots were created with R ggplot2 version 3.5.1 (*64*).

### Innervation volume and area quantification

To measure the three-dimensional (3D) volume, surfaces were created in Imaris from fluorescence signals (GFP or cherry) by defining a region of interest, with identical settings for AB-R and AB-L surfaces. The acquired volumes were recorded and statistically analyzed in Prism (GraphPad, version 10.2.3). Statistical significance was tested using the Wilcoxon paired-samples test for consistent differences between AB-R and AB-L measurements within individuals. The Mann-Whitney *U* test was performed to compare grouped AB-R with grouped AB-L values.

Fiji (version 2.14.0/1.54f) (*65*) freehand tool was used to select the biggest dorsoventral cross section of the AB (R72A10-lexA>lexAop-GFP signal) and the EB neuropils (CadN signal). The sum of AB-R and AB-L and the difference between AB-R and AB-L were normalized by dividing by EB cross section to account for variation in brain size between individuals. The normalized sum indicates general synaptic input to CX via AB innervations by afferent neurons ([AB-R + AB-L]/EB), and the normalized difference indicates the direction and strength of lateralization of synaptic input right versus left ([AB-R-AB-L]/EB). We defined phenotypic expressivity as the normalized difference.

Effect sizes were compared by computing rank-biserial correlation [R version 4.4.1 (*62*) and effectsize version 0.8.9 (*66*)], and plots were created in R [ggplot2 version 3.5.1 (*64*) and beeswarm version 0.4.0 (*67*)]. Statistical analysis was done in R using Wilcoxon rank sum tests with continuity correction to compare Fas2Mz507-Gal4 data with controls and Kruskal-Wallis and Dunn post hoc tests [rstatix version 0.7.2 (*68*)] to compare more than two groups.

### Clonal analysis

MultiColor FlpOut or Flybow 1.1B was crossed with Gal4-driver lines and maintained at 25°C. Offspring were heat shocked in a water bath at 37°C (10 min for MCFO and 1 hour for Flybow) at the late third instar stage.

### Pupal development

Pupal development was initially determined precisely to hours after pupal formation. These data were supplemented by pupae dissected in the early stages of development and their developmental stage approximated after imaging (*). Crosses and collected pupae were kept at 25°C.

To accurately time the pupae, pupae were collected within the first 0.5 hours after pupation (P0), and the subsequent time was counted as the hours APF. If the time from 0 to 0.5 hours before collection and the time for dissection to fixation are included, the hours APF are accurate to within 1 hour.

To approximate the hours APF, pupae were dissected after head evagination until before eye pigmentation, and the progress of EB development was used to assess the stage of development. The EB primordia develop in both hemispheres before fusing into a single rod-shaped neuropil at the midline in early pupal development. The edges of this structure begin to curve ventrally at 12 hours APF and gradually form the adult ring structure. We calculated the percentage of ring closure (EBC): The angle between the ventral edge of the gap and the dorsal edge of the EB canal was subtracted from the 360° of the completed adult ring and divided by 360° {closure (%) = [360° − gap°]/360° × 100}. We used the ring closure to estimate the hours APF on the basis of the ring closure data of our precisely staged pupae. Approximation of hours APF was based on linear regression (fig. S7; Prism version 10.2.3). EB closure and the portion of brains with segregated AB precursors in precisely staged pupae were plotted in Prism over hours APF. For more information, see the Supplementary Materials.

### Immunohistochemistry

Phosphate-buffered saline (PBS) buffer was prepared by dissolving 75.97 g of NaCl, 12.46 g of Na_2_HPO_4_, and 4.14 g of NaH_2_PO_4_ in 1 liter of water, and the pH was adjusted to 7.6. A 16% paraformaldehyde (PFA) stock solution was prepared from 0.8 g of PFA, 5 ml of water, and 35 μl of NaOH (1 M). Brains were dissected in PBS buffer and fixed in 4% PFA in PBS for 25 min at room temperature. Samples were washed four times in 0.3% Triton-X in PBS before blocking for 1 hour in 10% goat serum in PBS. Incubation with primary antibodies was performed at 4°C overnight. Samples were washed four times in 0.3% Triton-X in PBS followed by incubation with secondary antibodies overnight at 4°C. Samples were washed four times in 0.3% Triton-X in PBS before mounting in VECTASHIELD (Vector Laboratories) medium.

Primary antibodies used for this study were the rat anti-CadN extracellular domain (cells from DN-Ex no. 8, 1:10; Developmental Studies Hybridoma Bank, in-house production), mouse anti-Fas2 (1D4) intracellular domain (1:10, cells from Developmental Studies Hybridoma Bank, in-house production), rabbit anti-GFP (1:1000; Invitrogen), rabbit anti–HA (hemagglutinin) (Sigma-Aldrich), and rat anti-FLAG (Novus Biologicals). Secondary antibodies used for this study were goat anti-rabbit Alexa 488 (1:500, Invitrogen), goat anti-mouse highly cross-absorbed Alexa 488 (1:500, Invitrogen), goat anti-mouse highly cross-absorbed Alexa 568 (1:300, Invitrogen), goat anti-rabbit Alexa 568 (1:300, Invitrogen), and goat anti-rat Alexa 647 (1:500, Invitrogen). All antibodies were diluted in 10% goat serum in PBS at the indicated ratios. All resources are documented in table S1.

### Confocal acquisition and image analysis

Confocal imaging was performed using a Leica TCS SP5II microscope with a 20× oil immersion and a 40× water immersion objective. Images were processed with ImageJ and Imaris (Bitplane, version 9.3.1). Images, especially for SA^uni^ phenotype counting, were acquired at 400 Hz with a resolution of 512 by 512. Representative morphological data were acquired at 200 Hz and a resolution of 1024 by 1024.

### Satiety-dependent fructose drive

The flies were fasted for 30 hours at 29°C on 1% agarose gel. All flies were tested individually in the FlyPad (*69*) apparatus (Easy Behavior) following the protocol described in (*32*). The two electrodes were prepared with 3 μl of 1% agarose in 50 mM sugar solutions, one electrode with glucose and the other with fructose. During the experiments, the interactions of the flies with the presented sugar were recorded at each electrode for 1 hour. All the resources used are documented in table S1.

Preference indices were calculated from the interactions [performance index = (fructose − glucose)/(all interactions)]. The FlyPad output was preprocessed for further analysis in R. The results were plotted and statistically analyzed in Prism. The Wilcoxon paired-samples test was performed to test for consistent differences in the number of glucose/fructose interactions between individuals, and the Mann-Whitney *U* test was used to test for statistically significant effects between the performance indices per group.

### Single-session appetitive olfactory conditioning

The flies tested were raised on standard cornmeal food and a 12/12-hour light/dark cycle at 18°C. Fasting, training, and testing were performed at 23°C and 60% humidity. The training and tests were carried out in an elevator T-maze apparatus (*70*) following the protocol established in (*33*). The flies were fasted for 20 hours at humidity on 1% agar before training. For the training, the flies were first exposed to the conditioned stimulus − (CS−) without sugar reward (filter paper soaked in water) for 1 min. Then, the conditioned stimulus + (CS+) paired with a sugar reward (saturated sucrose solution on filter paper) was presented to the flies for 1 min. The odorant 3-octanol or 4-methylcyclohexanol was used alternately as CS+ or CS− in successive test runs with the same genotype. To prepare odor solutions, 8 μl of 3-octanol or 13 μl of 4-methylcyclohexanol was diluted in 8 ml of mineral oil. To test immediate memory performance, flies were directly transferred into the T-maze after training. To test 24 hours of memory expression, flies were kept on 1% agar for 24 hours after training. Testing was performed in complete darkness. The flies had 2 min to choose either the arm with CS+ or the arm with CS−. All the resources used are documented in table S1.

For each run, an index was calculated by dividing the difference between the number of flies in the CS+ arm and the number of flies in the CS arm of the T-maze by the total number of flies in both arms. The performance indices were calculated as the mean value of the indices for the reciprocal CS+/CS− training runs. Results were plotted and tested for statistically significant effects using Prism.

### Statistical analysis

Quantification and statistical analysis are described in the relevant Materials and Methods subsections above. Data S2 contains data sheet with all statistical tests, test parameters, test statistics, exact *P* values, and corrections described in the text or figures. The data shown in the figures can be found in data S3.
