# Supplementary material for: Sequential formation of Drosophila circuit asymmetry via prolonged structural plasticity
Source: Sci Adv. 2026 Mar 25;12(13):eaea6020. doi: 10.1126/sciadv.aea6020 (PMC13015903; doi:10.1126/sciadv.aea6020)
Supplement: Supplementary file 1 — Figs. S1 to S7 Table S1 Legends for data S1 to S3 [file sciadv.aea6020_sm.pdf]

Science Advances
AAAS

Supplementary Materials for

Sequential formation of *Drosophila* circuit asymmetry via prolonged structural plasticity

Johann W. Markovitsch *et al.*

Corresponding author: Thomas Hummel, thomas.hummel@univie.ac.at

Sci. Adv. **12**, eaea6020 (2026)
DOI: 10.1126/sciadv.aea6020

The PDF file includes:

Figs. S1 to S7
Table S1
Legends for data S1 to S3

Other Supplementary Material for this manuscript includes the following:

Data S1 to S3

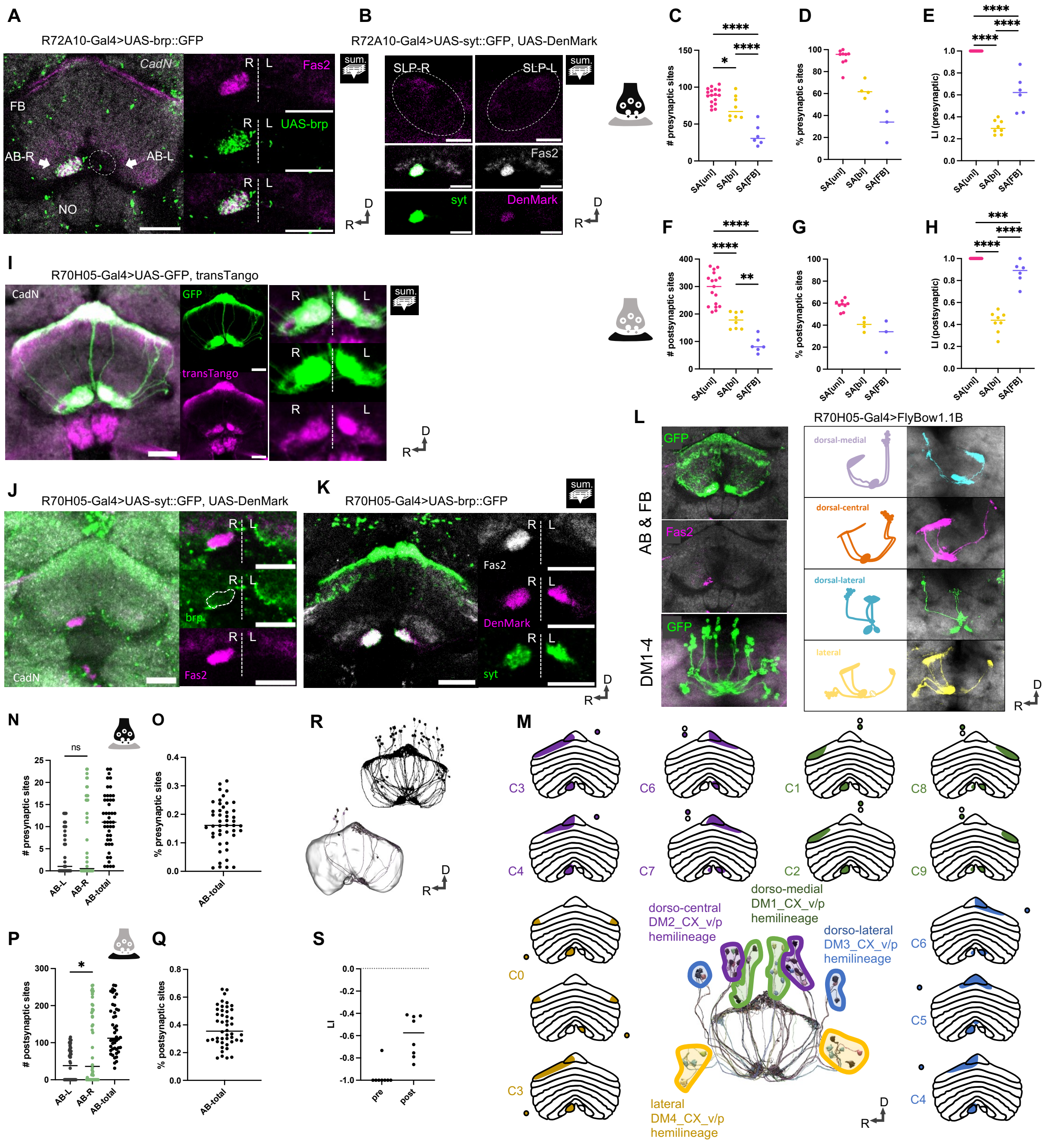

Fig. S1.

AB neuronal types differ in left-right asymmetric distributions of their synaptic connections. (A) Immunofluorescence detection of *UAS-brp::GFP* driven by *R72A10-Gal4* and anti-Fas2 (magenta) and antiCadN (grey) antibody staining. **(B)** Immunofluorescence detection of *UAS-syt::GFP* (green) and *UAS-DenMark* (magenta) driven by *R72A10-Gal4* and and Fas2 (grey). **(C)** Number of presynaptic sites in AB-R and AB-L per neuron for the three main SA afferent neuron types. SA^{uni} neurons have the highest number of presynaptic sites, SA^{FB} the lowest, one-way ANOVA revealed a significant difference between neuronal types in number of presynaptic sites, $F(2,28)=36.54$, $p=1.6*10^{-8}$. **(D)** Percent of presynaptic sites in AB-L and AB-R

compared to the total number of presynaptic sites of a neuron. While almost all presynaptic sites of SA^{uni} are located in the AB, SA^{bi} and SA^{FB} have a substantial number of presynaptic sites in the SLP or the dorsal FB respectively. **(E)** Lateralization Index per neuron by afferent type. Positive Indices indicate % of total AB presynaptic site that are more on the right (AB-R – AB-L)/(AB-all). All afferents show a right-directed lateralization of presynaptic site. SA^{bi} is the least lateralized afferent. one-way ANOVA revealed a significant difference between neuronal types in presynaptic lateralization (LI), $F(2,28) = 213.7$, $p = 1.1*10^{-17}$. **(F)** Number of postsynaptic sites per neuron in AB-R and AB-L for the three main SA afferent neuron types. One-way ANOVA indicated a significant difference between neuronal type in number of postsynaptic sites, $F(2,28) = 43.01$, $p=2.9*10^{-9}$. **(G)** Percent of postsynaptic sites in AB-L and AB-R compared to the total number of presynaptic sites of a neuron. All “afferents” receive substantial input in the AB neuropiles, SA^{uni} receives more input in AB-R than from the SLP. **(H)** Lateralization Index per neuron by afferent type. Positive Indices indicate % of total AB postsynaptic site that are more on the right (AB-R – AB-L)/(AB-all). Surprisingly, AB input to SA^{FB} comes almost exclusively from AB-R (One-way Anova, $F(2,28) = 218.2$,$p =8 .4*10^{-18}$). **(I)** Trans-Tango expression and GFP (green) driven by R70H05-Gal4 labels postsynaptic partners of vΔA neurons (magenta and CadN reference antibody staining (grey). **(J)** Immunofluorescence detection of *UAS-brp::GFP* driven by *R70H05-Gal4* and and Fas2 (magenta) and CadN (grey) antibody staining. **(K)** Immunofluorescence detection of *UAS-syt::GFP* (green) and *UAS-DenMark* (magenta) driven by *R70H05-Gal4* and and Fas2 (grey). **(L)** vΔA cell bodies are positioned in four clusters per hemisphere. Immunofluorescence detection from *UAS-GFP* (green, left panel / *UAS-Flybow1.1B* (right, panel: cyan, magenta, green, yellow) driven by *R70H05-Gal4* and Fas2 (left panel, magenta) and CadN antibody staining. **(M)** vΔA cell body clusters indicate their descendance from lineages DM1-4 (*24*). Neurons of each cluster follow a stereotyped projection pattern based on EM data (*18*). **(N, P)** Numbers of synaptic connections by vΔA in AB-L and AB-R and **(O, Q)** the % of total synaptic connections located in the ABs per vΔA neuron. Mann-Whitney U test reveals significant difference in number of postsynaptic sites of vΔA neurons in AB-L compared to AB-R, $U = 780$, $p = 0.0241$, but indicates that that numbers of presynaptic sites of vΔA neurons are most compatible with the hypothesis they are not differently distributed between AB-L and AB-R, $U = 916$, $p = 0.2397$. **(R)** Some vΔA neurons bilaterally innervate AB-L and AB-R **(S)** Normalized difference in pre- and postsynaptic sites between AB-R and AB-L in bilaterally projecting vΔA neurons (Lateralization Index, (R-L)/(R+L)) indicates mostly postsynaptic sites in AB-L. SA^{uni} in magenta, SA^{bi} in yellow and SA^{FB} in blue. Bars in plots indicate group medians. Significance thresholds are: *<0.05, **<0.01, ***<0.001, ****<0.0001. Pairwise comparisons by Tukey HSD test. For D and G only neurons of the right hemisphere were taken into account, because left SLP is not included in the hemibrain dataset.

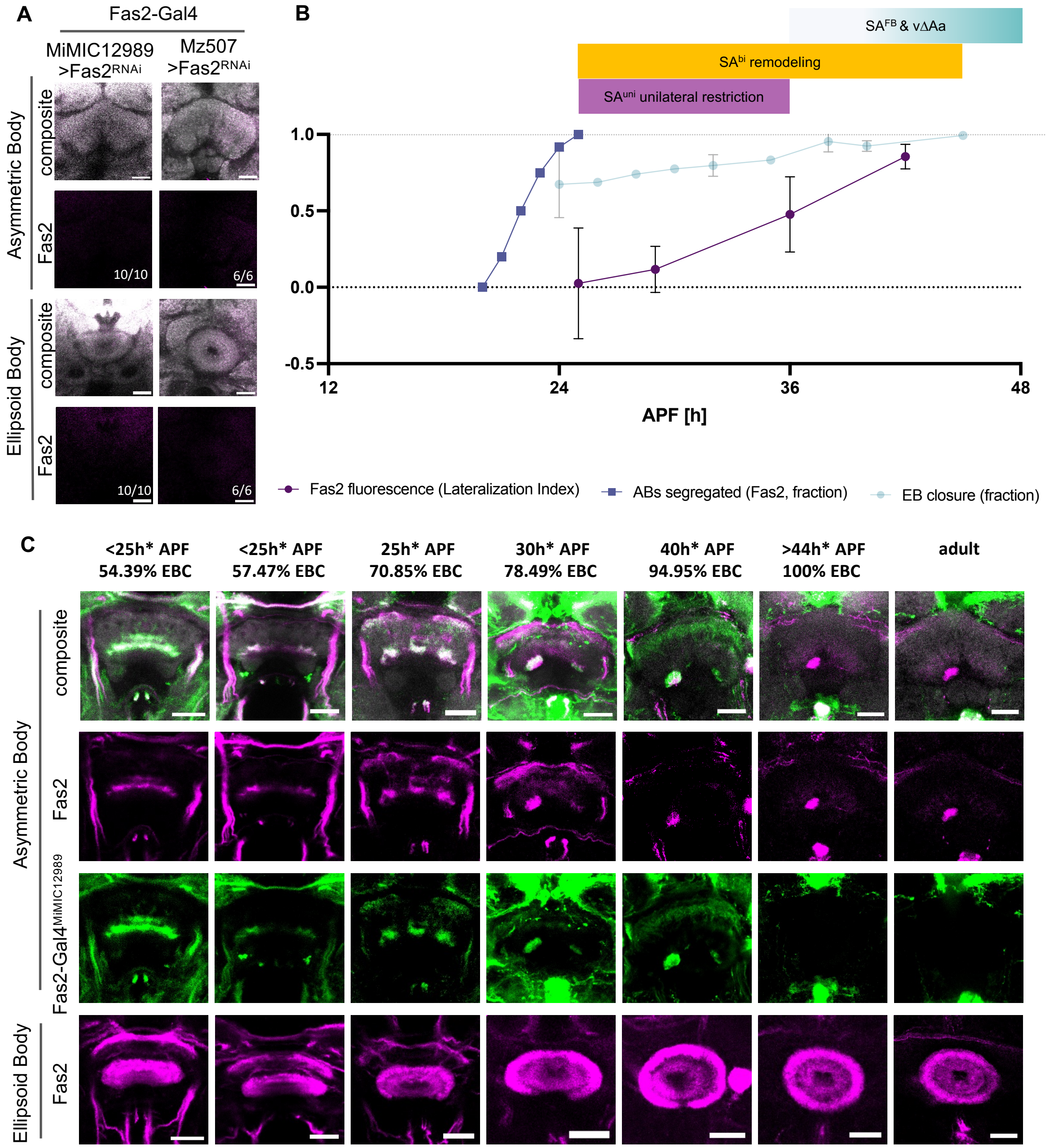

Fig. S2.

The expression of Gal4 under the control of the endogenous regulatory regions does not fully replicate the expression pattern of Fas2 in developing and adult ABs. (A) Following targeted knockdown driven under the control of the endogenous Fas2 regulatory regions, anti-Fas2 antibody staining is no longer detectable in the AB and neighboring CX neuropils. Immunodetection of anti-Fas2 (magenta) and anti-CadN (grey) antibody staining. **(B)** Timeline of AB development based on Fas2 expression (Fas2::GFP[397]). From 21h to 25h APF Fas2-positive AB primordia segregate from Fas2 positive layers in the developing ventral FB (rate of analysed brains with segregated AB precursors, blue squares). At 25h APF signal from anti-Fas2

immunodetection in AB-R and AB-L did not show a population-level directed lateralization, but detected fluorescence in AB-R became increasingly more intense compared to AB-L up to 42h APF (Lateralization Index (AB-R-AB-L)/(AB-R+AB-L), magenta dots (mean LI), bars indicate 95% Confidence Intervals). The maturation of the EB from an elongated oval to a circular shape occurs in the same time window of CX development, shows low variability and can be used as an approximation for the number of hours after pupal formation (fraction of closure measured by ventral gap (°) / 360, light blue dots (mean fraction, bars indicate 95% confidence intervals). **(C)** *Fas2* ^{MiMIC12989} *-Gal4* expression is restricted to the dorsal AB primordia. Endogenous expression of Fas2 appeared unaffected. Gal4 expression is absent in the adult AB. Immunodetection of *UAS-GFP* driven by *Fas2* ^{MiMIC12989} *-Gal4* (green) and anti-Fas2 (magenta) and anti-CadN (grey) antibody staining. The time after puparium formation (h APF) was approximated after imaging based on Ellipsoid Body (EB) closure (see methods). Immunodetection of *Fas2::GFP* ^{397} (green) and anti-Fas2 (magenta) and anti-CadN (grey) antibody staining. All scale bars, 20µm.

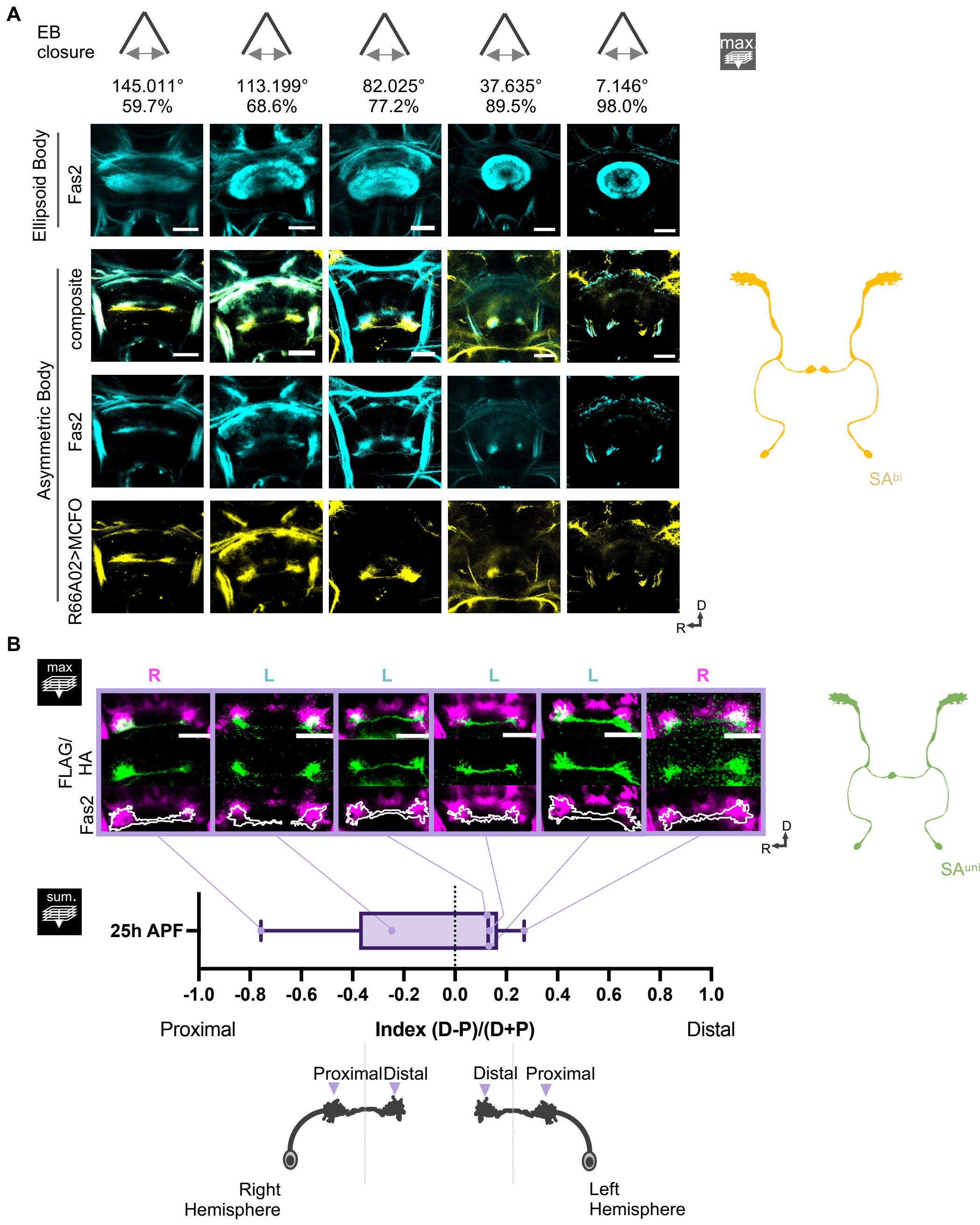

Fig. S3.

SA^{bi} neurons densely innervate the dorsal AB primordium, SA^{uni} neurons the ventral primordium. (A) Clonal analysis of SA^{bi} during pupal development based on immunodetection of *UAS-Flag/HA (MCFO)* driven by *R66A02-Gal4* in *hsFLP* induced mosaics (yellow), and anti-Fas2 antibody staining (cyan). Ellipsoid Body (EB) closure (%) indicates progress of pupal development. **(B)** At 25h SA^{uni} neurons innervate the ventral AB-R and AB-L primordia. Measurements from immunodetection of GFP signal from single cell clones (box plot). No consistent difference in innervation strength between proximal and distal innervations of the same neuron were detectable, while we found a tendency for the distal innervations to be denser.

Proximal and distal measurements were obtained from the same cell clones that were also analyzed for left–right lateralization in Fig. 5A. Immunodetection of *UAS-Flag/HA (MCFO)* by *R11F10-Gal4* in *hsFLP* induced mosaics (SA^{bi} clones in yellow, SA^{uni} clones in magenta) and anti-Fas2 antibody staining (cyan). All scale bars, 20µm.

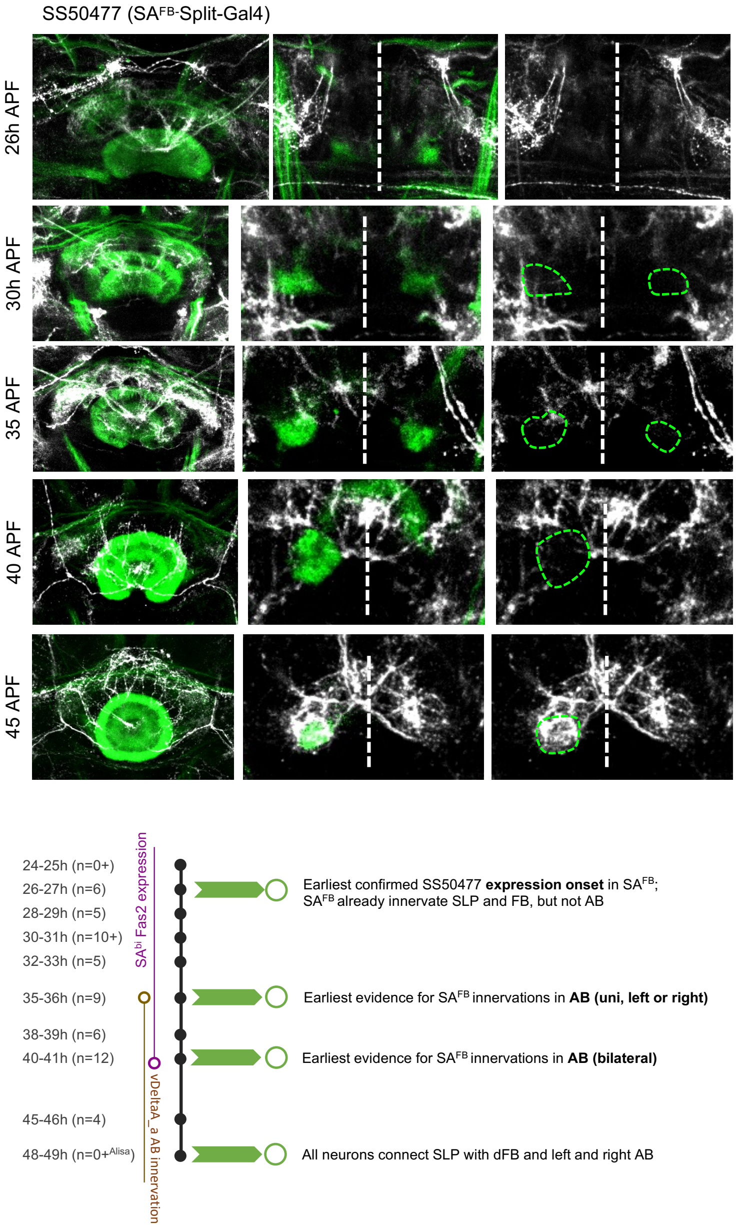

Fig. S4.

SA^{FB} innervation of the AB neuropiles starts after SA^{uni} and SA^{bi} axons have been remodeled. The timeline of AB connection development by SA^{FB} is based on the immunodetection of *UAS-FLAG* (grey), driven by *SS50477-Split-Gal4*, and anti-Fas2 antibody staining (green). Pupae were dissected at the indicated time (hours (h) after puparium formation, APF).

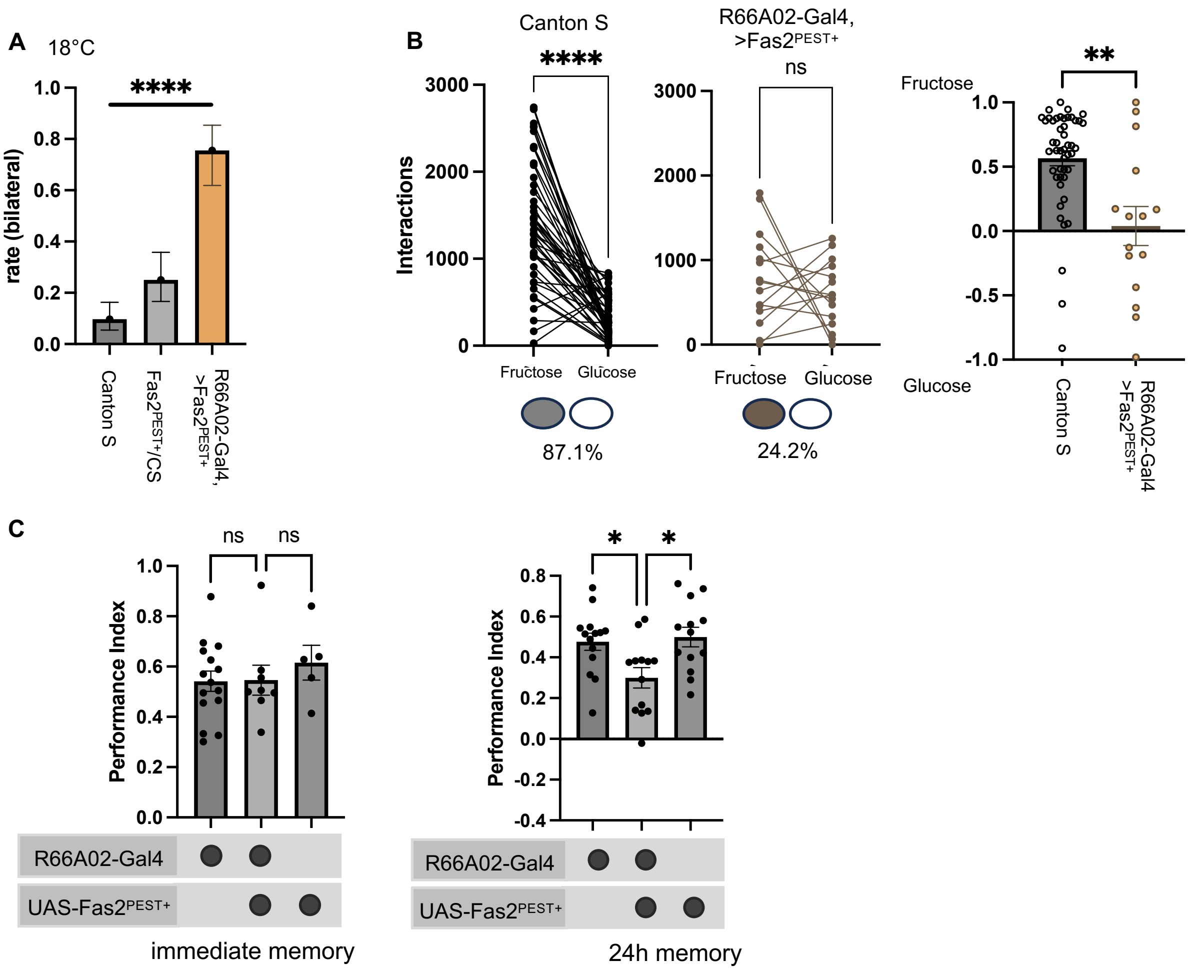

Fig. S5.

Bilateral $\mathrm{SA}^{\mathrm{uni}}$ projections alter adult Drosophila behavior. (A) The penetrance of the $\mathrm{SA}^{\mathrm{uni}}$ bilateral projection phenotype differs statistically significantly between flies expressing $\mathrm{Fas2}^{\mathrm{PEST+}}$ driven by *R66A02-Gal4* and control flies (Canton-S and *UAS-Fas2PEST+,* χ^2). **(B)** While wild-type flies (Canton-S) showed post-fasting preference for fructose over glucose, no consistent preference could be detected in flies expressing *Fas2PEST+* driven by *R66A02-Gal4* (Wilcoxon test for paired samples; Mann–Whitney U test). **(C)** Flies expressing Fas2PEST+ driven by R66A02-Gal4 exhibited immediate olfactory memory associated with a sugar reward. However, the expression of sugar-reward memory after 24 hours was statistically significantly lower than in the control groups (Kruskal–Wallis/Dunn test). The significance thresholds are: * < 0.05; ** < 0.01; *** < 0.001; **** < 0.001.

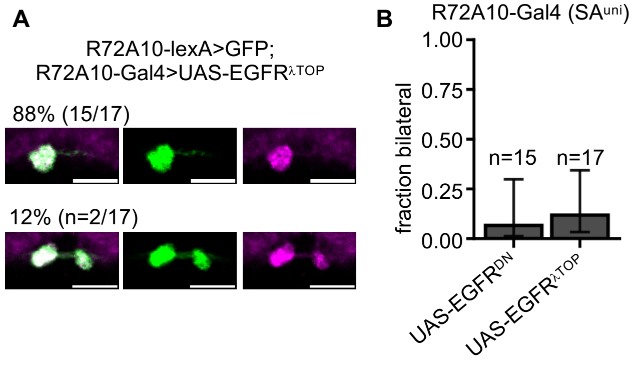

Fig. S6.

EGFR signaling does not stabilize bilateral SA^{uni} connections during axonal remodeling.
(A) Expression of a constitutively active form of EGFR (*UAS-EGFR$^{\lambda TOP}$*) in SA^{uni} did not increase the penetrance of bilateral SA^{uni} innervations compared to the expression of a dominant negative mutant (*UAS-EGFR^{DN}*). **(B)** Morphology of SA^{uni} innervations (green) of the AB expressing *UAS-EGFR$^{\lambda TOP}$* driven by *R72A10-Gal4*. Immunofluorescence detection of GFP driven by *R72A10-lexA* and anti-Fas2 (magenta) antibody staining. All scale bars, 20µm. Error bars indicate two-sided 95% confidence intervals for the single proportions. Significance thresholds are: *<0.05; **<0.01; ***<0.001; ****<0.001.

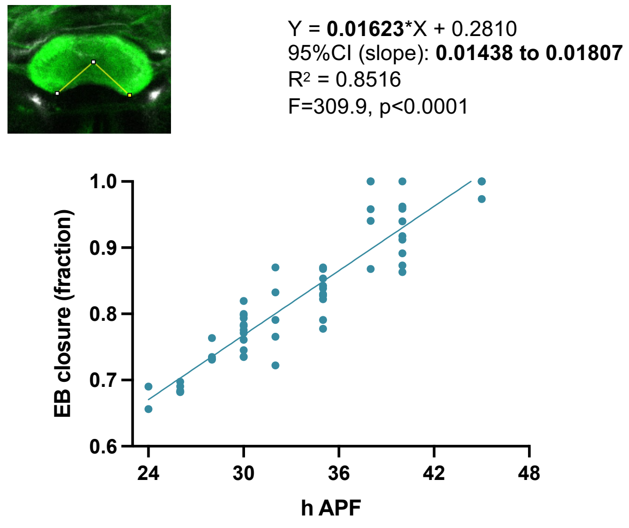

Fig. S7.

Correlation of EB closure and hours of pupal development. EB neurons gradually form a ring structure 20 to 44 hours after puparium formation. The angle formed by the gap and the center of the ring structure in % of the final complete ring (360°) are plotted over time. The measurements are from pupae of the *Fas2::GFP*^{397}transgene. The relationship between EB gap closure and h APF is almost linear in this time window. The formula for a simple linear regression model is given at the top of the figure.

Table S1: Resources used in this study, their source and catalogue number (Cat#) or research resource identifiers (RRID).

Resource	Source	ID
**Reagents - Immunostaining**		
Rabbit anti-GFP [1:1000]	Invitrogen / ThermoFisher Scientific	Cat# A-6455, RRID: AB_221570
Mouse anti-Fas2 [1:10]	In-house, cells from Developmental Studies Hybridoma Bank	Cat# 1D4, RRID: AB_528235
Rat anti-CadN [1:10]	In-house, cells from Developmental Studies Hybridoma Bank	Cat# DN-Ex #8, RRID: AB_528121
Rabbit anti-HA [1:2000]	Sigma-Aldrich / Merck	Cat# H6908, RRID: AB_260070
Rat anti-FLAG L5 [1:2000]	Novus Biologicals	Cat# NBP1-06712, N/A
Goat anti-Rabbit Alexa 488 [1:500]	Invitrogen / ThermoFisher Scientific	Cat# A-11008, RRID: AB_143165
Goat anti-Rabbit Alexa 568 [1:300]	Invitrogen / ThermoFisher Scientific	Cat# A-11011, RRID: AB_143157
Goat anti-Mouse highly-cross absorbed Alexa 488 [1:500]	Invitrogen / ThermoFisher Scientific	Cat# A-11029, RRID: AB_2534088
Goat anti-Mouse highly-cross absorbed Alexa 568 [1:300]	Invitrogen / ThermoFisher Scientific	Cat# A-11031, RRID: AB_144696
Goat anti-Rat 647 [1:500]	Invitrogen / ThermoFisher Scientific	Cat# A-21247, RRID: AB_141778
Normal Goat Serum	Sigma-Aldrich / Merck	Cat# G6767
NaCl	Carl Roth GmbH + Co. KG	Cat# 3957.1
KCl	Merck	Cat# 1.04936
Na_2HPO_4	Carl Roth GmbH + Co. KG	Cat# 4984.2
NaH_2PO_4	Carl Roth GmbH + Co. KG	Cat# K300.1
Triton X-100	Sigma-Aldrich / Merck	Cat# X100
Paraformaldehyde (PFA), pure, powder form	Carl Roth GmbH + Co. KG	Cat# 0964.1
NaOH, pellets	Merck	Cat# 1.06498
Vectashield	Vector Laboratories	Cat# H-1000
**Reagents - Satiety-dependent fructose drive**		
LE Agarose	Biozym Scientific GmbH	Cat# 840004
D-(+)-Glucose	Sigma-Aldrich / Merck	Cat# G7528
D-(-)-Fructose	Sigma-Aldrich / Merck	Cat# F0127
**Reagents - Single-Session Appetitive Olfactory Conditioning**		
Mineral oil	Sigma-Aldrich / Merck	Cat# M5904
4-methylcyclohexanol (98%)	Sigma-Aldrich / Merck	Cat# 153095
3-octanol (99%)	Sigma-Aldrich / Merck	Cat# 218405
Sucrose	Sigma-Aldrich / Merck	Cat# S0389
**Reagents - Injection empty-Gal4**		
pBPGUw plasmid in bacteria strain DB3.1 (50)	Addgene	Cat# 17575, RRID: Addgene_17575
QIAprep Spin Miniprep Kit	Qiagen	Cat# 27104
GelRed	Sigma Aldrich / Merck	Cat# SCT123
Ampicillin	Carl Roth GmbH + Co. KG	Cat# K029.4
Agar agar	Carl Roth GmbH + Co. KG	Cat# 5210.2
Tryptone	Carl Roth GmbH + Co. KG	Cat# 6952.2
Yeast extract	Carl Roth GmbH + Co. KG	Cat# 2363.2
Voltalef H3S Oil (Arkema)	Lehmann & Voss & Co	Cat# 21116230
Voltalef H10S (Arkema)	Lehmann & Voss & Co	Cat# 21116240
Phenol red solution	Sigma-Aldrich / Merck	Cat# P0290
Cello Glue	Tesa SE	Cat# 31.39-30
Hygiene cleaner with chlorine (DanKlorix)	Colgate-Palmolive	N/A
**Fly strains**		
D. melanogaster: R72A10-Gal4 (51)	Bloomington Drosophila Stock Center	Cat# 48306, RRID:BDSC_48306
D. melanogaster: R72A10-lexA *(50)*	Bloomington Drosophila Stock Center	Cat# 54191, RRID: BDSC_54191
D. melanogaster: R52H03-Gal4 (51)	Bloomington Drosophila Stock Center	Cat# 38849, RRID: BDSC_38849
D. melanogaster: R11F10-Gal4 (51)	Bloomington Drosophila Stock Center	No longer available, Flybase ID: FBal0249682
D. melanogaster: R66A02-Gal4 (51)	Bloomington Drosophila Stock Center	Cat# 39384, RRID: BDSC_39384
D. melanogaster: R66A02-lexA (50)	Bloomington Drosophila Stock Center	Cat# 52688, RRID: BDSC_52688
D. melanogaster: R16E08-Gal4 (51)	Bloomington Drosophila Stock Center	Cat# 39416, RRID: BDSC_39416
D. melanogaster: R70H05-Gal4 (51)	Bloomington Drosophila Stock Center	Cat# 39554, RRID: BDSC_39554
D. melanogaster: R38D01-Gal4 (51)	Bloomington Drosophila Stock Center	Cat# 49996, RRID: BDSC_49996

D. melanogaster: R14B01-Gal4 (51)	Bloomington Drosophila Stock Center	Cat# 48597, RRID: BDSC_48597
D. melanogaster: R14B01-lexA *(50)*	Bloomington Drosophila Stock Center	Cat# 52467, RRID: BDSC_52467
D. melanogaster: SS50477 (11)	Bloomington Drosophila Stock Center	No longer available, Flybase ID: FBco0001223
D. melanogaster: Fas2^{Mz507}-Gal4-Gal4	Benjamin Altenhein	Flybase ID: FBal0094026
D. melanogaster: Fas2^{MiMIC12989}-Gal4 (51)	Bloomington Drosophila Stock Center	Cat# 77831, RRID: BDSC_77831
D. melanogaster: hs-Flp, MCFO (52)	Bloomington Drosophila Stock Center	Cat# 64086, RRID: BDSC_64086
D. melanogaster: Flybow 1.1B (53)	Gift from Iris Salecker	Flybase ID: FBti0161267
D. melanogaster: 13x-lexAop-mCD8::GFP (Chr. II)	Gift, unknown	unkown
D. melanogaster: 13x-lexAop-mCD8::GFP (Chr. III)	Bloomington Drosophila Stock Center	Cat# 32203, RRID: BDSC_32203
D. melanogaster: 10x-UAS-mCD8::GFP	Bloomington Drosophila Stock Center	Cat# 32186, RRID: BDSC_32186
D. melanogaster: 10x-UAS-mCD8::RFP, 13x-lexAop-mCD8::GFP	Bloomington Drosophila Stock Center	Cat# 32229, RRID: BDSC_32229
D. melanogaster: UAS-Cherry	Bloomington Drosophila Stock Center	Cat# 27392, RRID: BDSC_27392
D. melanogaster: 10x-UAS-FLAG (52)	Bloomington Drosophila Stock Center	Cat# 62147, RRID: BDSC_62147
D. melanogaster: UAS-Fas2^{RNAi} (54)	Bloomington Drosophila Stock Center	Cat# 28990, RRID: BDSC_28990
D. melanogaster: UAS-Fas2^{PEST+} (55)	Gift from Corey S. Goodman	Flybase ID: FBal0066094
D. melanogaster: UAS-Fas2^{PEST-} (55)	Gift from Corey S. Goodman	Flybase ID: FBtp0000962
D. melanogaster: UAS-intra-Fas2^{PEST-}::YFP (22)	Gift from Akinao Nose	Flybase ID: FBal0241547
D. melanogaster: UAS-extra-Fas2^{PEST-}::YFP (22)	Gift from Akinao Nose	Flybase ID: FBal0241546
D. melanogaster: UAS-unc-5^{RNAi} (54)	Bloomington Drosophila Stock Center	Cat# 33756, RRID: BDSC_33756
D. melanogaster: UAS-NetB^{RNAi} (54)	Bloomington Drosophila Stock Center	Cat# 25861, RRID: BDSC_25861
D. melanogaster: UAS-hid^{14}	Gift from John R. Nambu	N/A
D. melanogaster: Nrg^{849}	Bloomington Drosophila Stock Center	Cat# 35827, RRID: BDSC_35827
D. melanogaster: UAS-syt::GFP	Bloomington Drosophila Stock Center	Cat# 6925, RRID: BDSC_6925
D. melanogaster: UAS-DenMark::cherry (58)	Bloomington Drosophila Stock Center	Cat# 33062, RRID: BDSC_33062
D. melanogaster: UAS-brp::GFP (S. Sigrist)	Bloomington Drosophila Stock Center	Cat# 36291, RRID: BDSC_36291
D. melanogaster: trans-Tango (59)	Bloomington Drosophila Stock Center	Cat# 77124, RRID: BDSC_77124
D. melanogaster: nysb-GRASP (60)	Bloomington Drosophila Stock Center	Cat# 64315, RRID: BDSC_64315
D. melanogaster: t-GRASP (61)	Bloomington Drosophila Stock Center	Cat# 79039, RRID: BDSC_79039
D. melanogaster: UAS-EGFR*[lambdaTOP] (56)*	Bloomington Drosophila Stock Center	Cat# 59843, RRID: BDSC_59843
D. melanogaster: UAS-EGFR[DN] *(57)*	Bloomington Drosophila Stock Center	Cat# 5364, RRID: BDSC_5364
D. melanogaster: lexAop-Cherry	N/A	N/A
D. melanogaster: brp(RSRT.STOP)::V5-T2A-lexA, 20xRSR[PEST] (35)	Bloomington Drosophila Stock Center	Cat# 55756, RRID: BDSC_55756
D. melanogaster: tub-Gal80[ts] (27)	Bloomington Drosophila Stock Center	Cat# 7108, RRID: BDSC_7108
D. melanogaster: Canton S	Bloomington Drosophila Stock Center	Cat# 64349, RRID: BDSC_64349
D. melanogaster: nanos-phiC31,attP2	Bloomington Drosophila Stock Center	Cat# 99002, RRID: BDSC_99002
D. melanogaster: empty-Gal4 (attP2)	this paper	N/A
**Software**		
Fiji (ImageJ)	Open-source / National Institutes of Health (NIH)	RRID: SCR_002285
R / RStudio	Open-source / Posit PBC	RRID: SCR_001905 / SCR_000432
GraphPad Prism	GraphPad Software, Inc.	RRID:SCR_002798
IMARIS	Oxford Instruments plc	RRID: SCR_007370

Data S1. (separate file)

Data sheet showing the parental genotypes of crosses between different fly strains and the genotypes of the F1 generation offspring.

Data S2. (separate file)

Data sheet with statistical tests, parameters, test statistics, p-values, and confidence intervals.

Data S3. (separate file)

Data sheet containing all data presented graphically in the figures of this study. Laboratory-internal IDs are provided for data based on microscope images.
